# Enabling High Strength and Toughness Polyurethane through Disordered‐Hydrogen Bonds for Printable, Recyclable, Ultra‐Fast Responsive Capacitive Sensors

**DOI:** 10.1002/advs.202405941

**Published:** 2024-10-14

**Authors:** Xingbao Chen, Shiwei Zhao, Anqian Yuan, Silong Chen, Yansheng Liao, Yuan Lei, Xiaowei Fu, Jingxin Lei, Liang Jiang

**Affiliations:** ^1^ State Key Laboratory of Polymer Materials Engineering Polymer Research Institute of Sichuan University Chengdu 610065 China

**Keywords:** alcohol soluble recycling, disordered‐hydrogen bonds, high strength and toughness, sensitive capacitive sensors, ultra‐fast responsive

## Abstract

The rapid advancement of smart, flexible electronic devices has paralleled a surge in electronic waste (e‐waste), exacerbating massive resource depletion and serious environmental pollution. Recyclable materials are extensively investigated to address these challenges. Herein, this study designs a unique polyurethane (SPPUs) with ultra‐high strength up to 60 MPa and toughness of 360 MJ m^−3^. This synthetic SPPUs can be fully recycled at room temperature by using green solvents of ethanol. Accordingly, the resultant SPPU‐Ni composites, created by mixing the ethanol‐dissolved SPPUs solution with nickel (Ni) powder, effectively combine the flexibility and recyclability of SPPUs with the electrical conductivity of the nickel filler. Notably, this work develops the printable capacitive sensors (PCBS) through transcribing the paste of SPPUs‐Ni slurry onto PET film and paper using screen‐printing technology. The devised PCBS have fast response time ≈50 ms, high resolution, and multiple signal recognition capabilities. Remarkably, SPPUs and Ni powder can be fully recycled by only dissolving the waste PCBS in ethanol. This work offers a sustainable solution to the growing e‐waste problem in recyclable flexible electronics.

## Graphical abstract

The environment‐friendly capacitive sensors are designed to address the problems of difficult recycling and high recycling cost of the current flexible sensor devices. This devised sensor features an extremely fast response time ≈50 ms, high resolution, and multiple signal recognition capabilities, providing a stable signal output even at a frequency of 5 Hz.

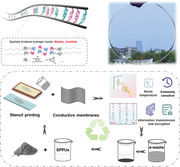

## 1 Introduction

Flexible electronics have emerged as a pivotal element in the evolution of intelligent, active human‐computer interaction systems.^[^ ^1^, ^2^, ^3^ ^]^ These electronics enable interactive information conversion and recognition processes, transforming external signals into the visualized electrical responses.^[^ ^4^, ^5^, ^6^ ^]^ For example, electronic skin that mimics the sensory capabilities of human skin, capable of detecting various external stimuli and identifying materials through pressure recognition, represents a significant advancement.^[^ ^7^, ^8^ ^]^ At the heart of these advancements lies the utilization of polymer matrix with their versatile mechanical properties and ease of functionalization benefiting from the seamless integration of their inherent flexibility and electrical conductivity, surpassing the capabilities of conventional rigid sensing systems.^[^ ^9^, ^10^ ^]^ This distinct advantage positions polymeric flexible electronics as a superior alternative, paving the way for novel applications in human‐computer interaction.

Despite polymeric flexible electronics play an indispensable role in their centrality to the burgeoning field, there are still challenges to face the inherent stability limitations compared to their inorganic counterparts, resulting in devices with constrained lifespans.^[^ ^11^ ^]^ These devices are damaged or deteriorated during use and are designated as electronic waste (e‐waste), which accumulates in landfills or natural environments for long periods of time, posing a serious threat to natural ecosystems.^[^ ^12^ ^]^ Recent advancements in those flexible electronics have predominantly concentrated on enhancing device stability and sensitivity.^[^ ^5^, ^13^ ^]^ Nonetheless, the aspect of component disassembly and the efficacious recycling of these devices have received minimal focus. Flexible electronic devices, which consist of polymers, conductive materials, and electronic components, are intended for a wider range of applications than traditional rigid electronic devices. These flexible electronics are typically tailored for single‐use in a variety of applications such as health diagnostics, soft robotics, and smart packaging, and potentially unintentionally contributing to the proliferation of e‐waste.^[^ ^14^, ^15^ ^]^ Presently, a significant proportion of these devices are non‐recyclable, exacerbating the accumulation of e‐waste. Although there have been isolated efforts toward recycling flexible electronic devices, these approaches have typically involved simple re‐mixing processes that fail to effectively separate the functional fillers and the polymeric substrates. This inadequacy underscores the pressing need for a method capable of efficiently disentangling these components while preserving the integrity of the recycled materials.^[^ ^16^, ^17^ ^]^ Addressing this challenge is crucial for reducing environmental pollution and conserving resources, making a significant step forward in the sustainable development of recyclable flexible electronics.

Dynamic covalent bond that is recognized for its unique ability to balance the mechanical properties and recyclability for crosslinked polymers, has acquired considerable attention in recent research.^[^ ^18^ ^]^ Lately, considerable effort has been made to employ various dynamic covalent bonds including hindered urea,^[^ ^19^, ^20^ ^]^ disulfide,^[^ ^21^, ^22^ ^]^ Diels‐Alder (DA) reactions^[^ ^23^ ^]^ and among others into to cross‐linked polymeric flexible electronics for recycling. As the vast portion of the dynamic covalent bonding is activated at elevated temperatures and high pressures are required for structural remodeling, the recovery of materials under milder conditions is not feasible. Additionally, these processes do not allow for the separation of substrate materials and fillers, merely facilitating their remixing. Illustratively, Obed J. Dodo and colleagues pioneered the synthesis of self‐healing and hot‐pressing reprocessable matrix materials. It was utilized a multifaceted dynamic network based on Thiol‐Michael exchange, Diels‐Alder reaction, and quadrupole hydrogen bond chemistries, incorporating multi‐walled carbon nanotubes (MWCNTs) to achieve a stretch‐sensing flexible polymer.^[^ ^17^ ^]^ However, this method encounters substantial limitations of material property degradation at elevated temperatures and inadequate recycling efficiency, which fail to meet practical recycling requirements. For addressing above challenges, Gou et al. synthesized a type of furan‐containing polyurethane (FPU) using specific DA adducts of 2,5‐furandimethanol and bismaleimide as the crosslinking unit.^[^ ^24^ ^]^ Remarkably, this FPU maintained a stable cross‐linking structure at ambient temperatures with a significant reduction in cross‐linking density at the reversible DA temperature (*T*_rDA_) in a state sustainable for extended periods. This characteristic enabled the dissolution of FPU in common organic solvents such as chloroform, acetone, or tetrahydrofuran at room temperature after high‐temperature treatment, facilitating the separation and recovery of electronic devices and substrate materials. Whereas these aforementioned methods simplify material recycling and separation, the necessary use of high temperatures and potentially toxic solvents during the recycling process contradicts the principles of green and environmental protection, underscoring the need for further advancements in this field.^[^ ^25^ ^]^

Linear polymers are garnering significant interest among researchers due to their unique solubility in specific solvents at ambient temperatures. Zhong et al. devised a series of novel nanodroplet‐crosslinked hydrogels by utilizing 2‐hydroxyethyl acrylate (HEA) and liquid metal (LM). This innovative approach integrated sonicated LM suspensions and silver flakes during the polymerization of HEA to produce conductive polymer films.^[^ ^26^ ^]^ These films exhibited outstanding tensile properties and electrical conductivity, reaching up to 1800 S cm^−1^. Notably, they can degrade in a NaOH solution at room temperature with the recovery efficiency of the fillers approaching 90%. Similarly, Guo et al. developed polyvinyl alcohol (PVA)‐based materials by using LM and iron (Fe) particles as fillers. These materials demonstrated exceptional remote self‐healing capabilities under a magnetic field, along with water‐degradability and thermal transfer printing functionalities.^[^ ^27^ ^]^ Such attributes rendered these proposed materials, prepared by the above methods, highly suitable for flexible sensors, boasting excellent tensile strength and favorable electrical conductivity. However, the application of LM presents an intractable problem of leakage,^[^ ^28^ ^]^ which is primarily attributed to its significantly higher surface tension compared to conventional organic materials and its weak interaction with substrate materials. Leakage of LM not only risks performance degradation but may also compromise the operation of adjacent circuits.^[^ ^29^ ^]^ Furthermore, the water degradability of these materials presents an additional concern. Devices in everyday use are inevitably subject to humidity fluctuations and, at times, direct water exposure. Insufficient water resistance can severely affect the operational stability of equipment, thereby imposing stringent requirements on the working environment.^[^ ^11^ ^]^

**Figure 1:**
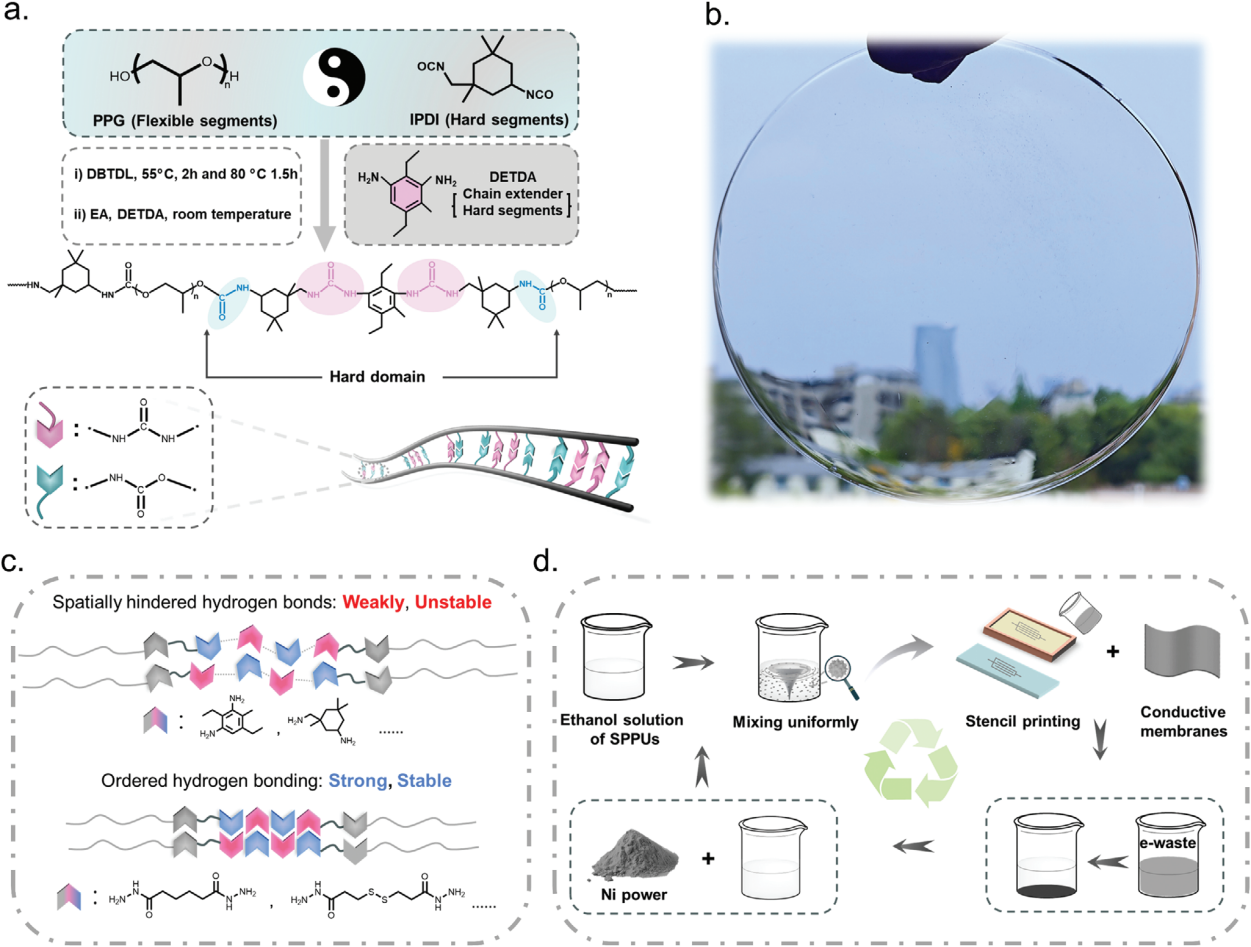
a) Schematic diagram of the synthesis of SPPUs and its hydrogen bonding interactions. b) Light transmittance of SPPUs (the inset is the digital picture of SPPU‐3). c) Synergistic effect of disordered hydrogen bonds and benzene rings. d) Schematic diagram of the preparation and recovery process of SPPU‐3_1/xNi_ (Ethanol as solvent at room temperature).

To address the aforementioned obstacles, this work aims to design and synthesize high‐strength, tough, and alcohol‐soluble recyclable polyurethanes (SPPUs), proposed as substrate materials for flexible sensors (**Figure** **1**a). Inspired by the multifunctionality of human skin, we target the development of printable capacitive‐based sensors (PCBS) characterized by high sensitivity, enhanced water resistance, and rapid, alcohol‐facilitated separable recycling. Our strategy involves a straightforward synthetic route for SPPUs using commercially available raw materials, a meticulously defined molecular structure (hindered‐hydrogen bonds), and the selection of functional fillers that are easily separable due to the significant density differential between SPPUs and nickel (Ni) powder. As substrate materials, SPPUs achieve an optimal balance between flexibility, strength, and toughness, exhibiting a tensile strength up to 60 MPa, an elongation at break ≈1500%, and a toughness ≈360 MJ m^−^^3^, surpassing even the toughest known spider silk at 354 MJ m^−^^3^. The unique hindered‐hydrogen bonds in SPPUs aligns with contemporary principles of green chemistry, allowing waste SPPUs to be dissolved in the environmentally benign solvent ethanol (derived from crop fermentation) to facilitate material recycling. This property is ascribed to the synergistic effect of disordered‐hydrogen bonds and benzene rings as shown in Figure 1c. The disordered‐hydrogen bonds mentioned are formed by hard segments containing spatial site resistance, forming a strong and weak combination between the hydrogen bond donor and the hydrogen bond acceptor.

Exploiting the exceptional alcohol solubility of SPPUs, we dissolved them and doped with Ni powder to fabricate flexible, filler‐ and substrate‐material‐separable films with excellent flexibility (shown in Figure 1d). The incorporation of Ni powder not only imparts electrical conductivity but also significantly accelerates the dissolution process, enabling the rapid separation of substrate from Ni powder within minutes using minimal solvent volumes, achieving nearly 100% separation efficiency. Crucially, both recycled Ni powder and SPPUs maintained their original properties after multiple recycling cycles. Given these advantages, we devised SPPU‐3_1/xNi_ paste for circuit printing. Circuits were screen printed onto PET films, SPPU films and paper, yielding highly sensitive, multifunctional flexible touch sensors that can emulate human skin functionalities, including information transmission, object identification, and water leakage detection. The separation and recycling of raw materials are achieved through ethanol dissolution of the discarded electronic waste, heralding the prospect of perpetual recycling for flexible electronic devices. Our work underscores the innovation of high‐quality, flexible, and recyclable transient electronics, potentially advancing applications in fields such as flexible robotics, human‐computer interaction, and electronic skin, thus marking a significant contribution to the advancement of polymeric flexible electronic technologies.

## 2 Results and Discussion

### 2.1 Material Synthesis and Structural Characterization

**Figure 2:**
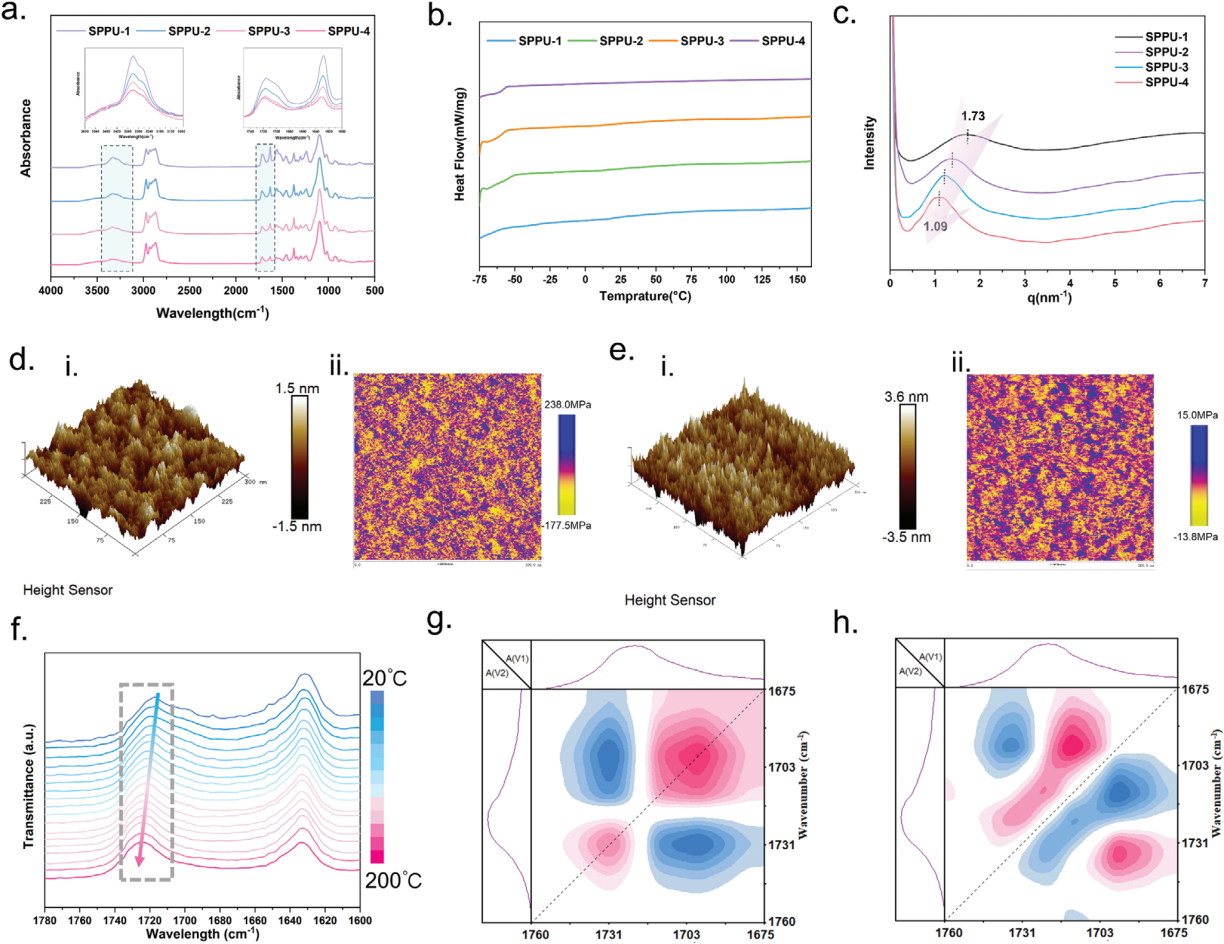
a) FTIR spectrogram of SPPUs (the inset is a partially enlarged view of 3600–3060 and 1750–1600 cm^−1^). b) DSC profile of SPPUs. c) SAXS profile of SPPUs. AFM height (i) and modulus (ii) maps of a 300 × 300 nm^2^ region of d) SPPU‐1 and e) SPPU‐3. f) 1D in situ FTIR of SPPU‐3. g) The synchronous and h) asynchronous 2D‐COS spectra calculated for the 1700–1675 cm^−1^ band of SPPU‐3. Pink and blue areas indicate positive and negative values, respectively, and signal intensity increases with color intensity.

The synthesis process of SPPUs was detailed in Figure 1a, with raw material compositions provided in Table S1, Supporting Information. The addition reaction of polypropylene glycols (PPGs) of varying molecular weights with isophorone diisocyanate (IPDI) in a molar ratio of 1:2 yielded prepolymers, which were then chain‐extended using stoichiometric amounts of diethyltoluenediamine (DETDA) to form highly transparent SPPUs samples (designated as SPPU‐X, where X = 1 to 4). This strategy leverages synergistic effects between strong hydrogen bonding and spatial resistance, endowing these SPPUs with high strength and toughness, while ensuring their alcohol solubility for sustainable, non‐polluting recycling. Additionally, the moderate reactivity of DETDA and minimal solvent requirements throughout the synthesis process make this method suitable for upscaling and molecular weight enhancement. To elucidate the structural advantages of SPPUs, we replaced PPG's methyl‐containing soft segments with crystalline polyethylene glycol (PEG) and side‐chain‐free polytetramethylene glycol (PTMG), yielding SPEU‐3 and SPTU‐3, respectively. Furthermore, we adjusted the hard segments by substituting aromatic DETDA with linear ethylenediamine (EDA) and cyclic ester‐based isophoronediamine (IPDA), producing control samples SPPU‐3_EDA_ and SPPU‐3_IPDA_, with their compositions detailed in Table S2, Supporting Information. We compared the properties of different samples for investigating the effect of changes in soft and hard segments on hydrogen bonding, crystallization and mechanical properties. The adoption of diamines as chain extenders other than conventional polyol extenders, enriches the molecular structure of SPPUs with dense urea (‐NH─CO─NH─) and urethane (─NH─COO─) functionalities, promoting microphase separation.^[^ ^30^ ^]^ A thorough thermogravimetric analysis (TGA) was performed to ascertain the samples' thermal stability and solvent residual status (Figure S1, Supporting Information).^[^ ^31^ ^]^ Detailed Fourier‐transform infrared spectroscopy (FTIR) analyses was used to reveal characteristic structure of the SPPUs and its control samples. As depicted in **Figure** **2**a, the absorption peak of SPPUs at 1090 cm^−1^ corresponds to the in‐plane vibration of the benzene ring C─H due to the presence of two polar urea (─NH─COO─) moieties in the vicinity of the benzene ring.^[^ ^32^ ^]^ The peaks at 1628, 1458, and 1373 cm^−1^ are attributed to the vibrations of benzene ring skeleton. These absorption peaks are present in different SPPUs samples, confirming the uniform incorporation of DETDA into the molecular structure of SPPUs. The absorption peaks at 1715 and 1240 cm^−1^ correspond to the stretching vibration peaks of C═O and C─O in urethane moieties, respectively.^[^ ^32^, ^33^ ^]^ Detailed investigation in the inset of Figure 2a indicates a shoulder‐like broad peak at 1689 cm^−1^, which becomes more pronounced with increased hard segment content, belonging to the stretching vibration of C═O in the urea group.^[^ ^30^ ^]^ The FTIR results indicate that all the SPPUs contain absorption peaks of urea (─NH─CO─NH─), urethane (─NH─COO─), and benzene ring moieties, confirming the successful synthesis of SPPUs. The control samples' FTIR spectra, shown in Figure S2, Supporting Information, underscore significant differences in the benzene ring and carbonyl group absorption peaks. The characteristic peaks of benzene rings are non‐existent in the control samples, and differences in the absorption peaks ≈1710 and 1630 cm^−1^ are attributed to the varied hydrogen bonding dynamics induced by different diamines.^[^ ^34^ ^]^

To elucidate the aggregation state structure of segmented SPPUs and their physical behavior under thermal transitions, a comprehensive study were conducted by X‐ray diffraction (XRD) and differential scanning calorimetry (DSC), alongside comparative analyses with control samples^[^ ^35^ ^]^ (Figure 2b, Figures S3 and S4, Supporting Information). The XRD patterns revealed a predominantly amorphous structure for SPPUs, characterized by a broad peak at 2θ from 10° to 30°. This amorphous nature was further corroborated by the absence of crystallization peaks except for SPPU‐3_PEG_, which exhibited distinct crystallization signals at 2θ = 19.6° and 23.8°, attributable to PEG crystallization.^[^ ^34^ ^]^ Consistent with the XRD results, the DSC curves showed only the glass transition temperature (*T*_g_) without the melting peaks, affirming the amorphous state of these SPPUs. This observation was visually supported by the high transparency of SPPUs, with transmittance rates exceeding 90%, implying their amorphous structure at the macroscopic level (Figures 1b and S5, Supporting Information). The phase morphology of SPPUs was further investigated using small‐angle X‐ray scattering (SAXS), which validated the presence of microphase‐separated structures through broad scattering peaks (Figure 2c).^[^ ^36^ ^]^ These results suggested that hydrogen bonding and the rigidity of hard segments facilitated their aggregation, serving as nano‐scale reinforcement and physical cross‐linking points. While PPG as a soft segment modulates the elastomers' strength and toughness.^[^ ^33^, ^37^ ^]^ Calculations based on SAXS data revealed that the average distances between hard regions in SPPUs were 3.63 nm for SPPU‐1, 4.62 nm for SPPU‐2, 5.24 nm for SPPU‐3, and 5.76 nm for SPPU‐4, respectively, with increasing molecular weight of the soft segments reducing the content of hard regions^[^ ^30^ ^]^ (Equation S1, Supporting Information). Atomic force microscopy (AFM) further visualized these structural variations, showing a progressive increase in the distance between hard segments correlating with a decrease in hard segment content from SPPU‐1 to SPPU‐4^[^ ^37^ ^]^ (Figures 2d,e and S6, Supporting Information). This variation corresponds to the SAXS‐derived data and revealed a structural evolution toward less densely packed hard segments with increasing soft segment molecular weight. To delve into the hydrogen bonding dynamics within SPPUs, in situ FTIR was employed in Figures 2f and S7, Supporting Information. All samples exhibited the change in peak position near 1720 cm^−1^ and the variation in peak intensities ≈1695 cm^−1^ with the increase in temperature. Taking SPPU‐3 as an example for detailed analysis (Figure 2f), with the gradual increase in temperature from 20 to 200 °C, the C═O absorption peak in SPPU‐3 shifted from 1717 to 1724 cm^−1^ without a change in intensity, attributed to the gradual dissociation of the hydrogen bonding leading to the red shift of the C═O absorption peak.^[^ ^34^, ^37^ ^]^ Additionally, the broad peak near 1695 cm^−1^ progressively disappeared with increasing temperature, indicating a sequential and synergistic relationship among the absorption peaks during the temperature rise.^[^ ^34^ ^]^ Subsequently, 2D infrared correlation spectra (2D‐COS) in the 1760–1670 cm^−1^ band of SPPU‐3 were calculated to analyze the detailed changes in hydrogen bonding during the warming process (Figure 2g,h). Two main auto‐peaks, namely (1736 cm^−1^, 1736 cm^−1^) and (1697 cm^−1^, 1697 cm^−1^), were observed in the synchronous spectrum, and two main cross‐peak, namely (1736 cm^−1^, 1697 cm^−1^) and (1714 cm^−1^, 1697 cm^−1^), was observed in the asynchronous. Following Nado's rule,^[^ ^38^ ^]^ it can be concluded that the sequence of change of absorption peaks with increasing temperature is 1736 cm^−1^ followed by 1697 cm^−1^. The absorption peaks of C═O are blue‐shifted in the presence of hydrogen bonding and red‐shifted as the hydrogen bonds dissociate with increasing temperature. Researchers have identified the absorption peaks near 1730 cm^−1^ as attributable to the free C═O groups, with the hydrogen bonded ordered C═O absorption peaks appear in the lower wave number range and hydrogen‐bonded disordered C═O groups in the moderate range. The strong absorption peak of the benzene ring in the SPPUs partially covered the absorption peak of hydrogen‐bonded ordered C═O. Therefore, in this work, the absorption peak near 1730 cm^−1^ was considered to belong to the free C═O, the absorption peak near 1714 cm^−1^ to the hydrogen bonded disordered C═O, and the absorption peak at 1697 cm^−1^ to the hydrogen bonded ordered C═O. The sensitivity of the absorption peak of the free C═O to the temperature fluctuation was weaker than that of the ordered one. According to in‐suit FTIR spectra and 2D‐COS, it was concluded that the absorption peak at 1717 cm^−1^ (hydrogen bonded disordered C═O) first shifted to higher wave numbers, accompanied by the gradual red shift of the peak at 1717 cm^−1^. The absorption peak of hydrogen‐bonded ordered C═O dissociated only at higher temperatures, corresponding to the shift of the absorption peak at 1695 cm^−1^. These results convincingly demonstrated the existence of hydrogen‐bonded interactions in SPPUs and illustrated the sequential changes of different hydrogen‐bonded interactions with temperature variations, providing a theoretical guide for SPPUs in varying usage temperatures.

### 2.2 Mechanical Properties of SPPUs

**Figure 3:**
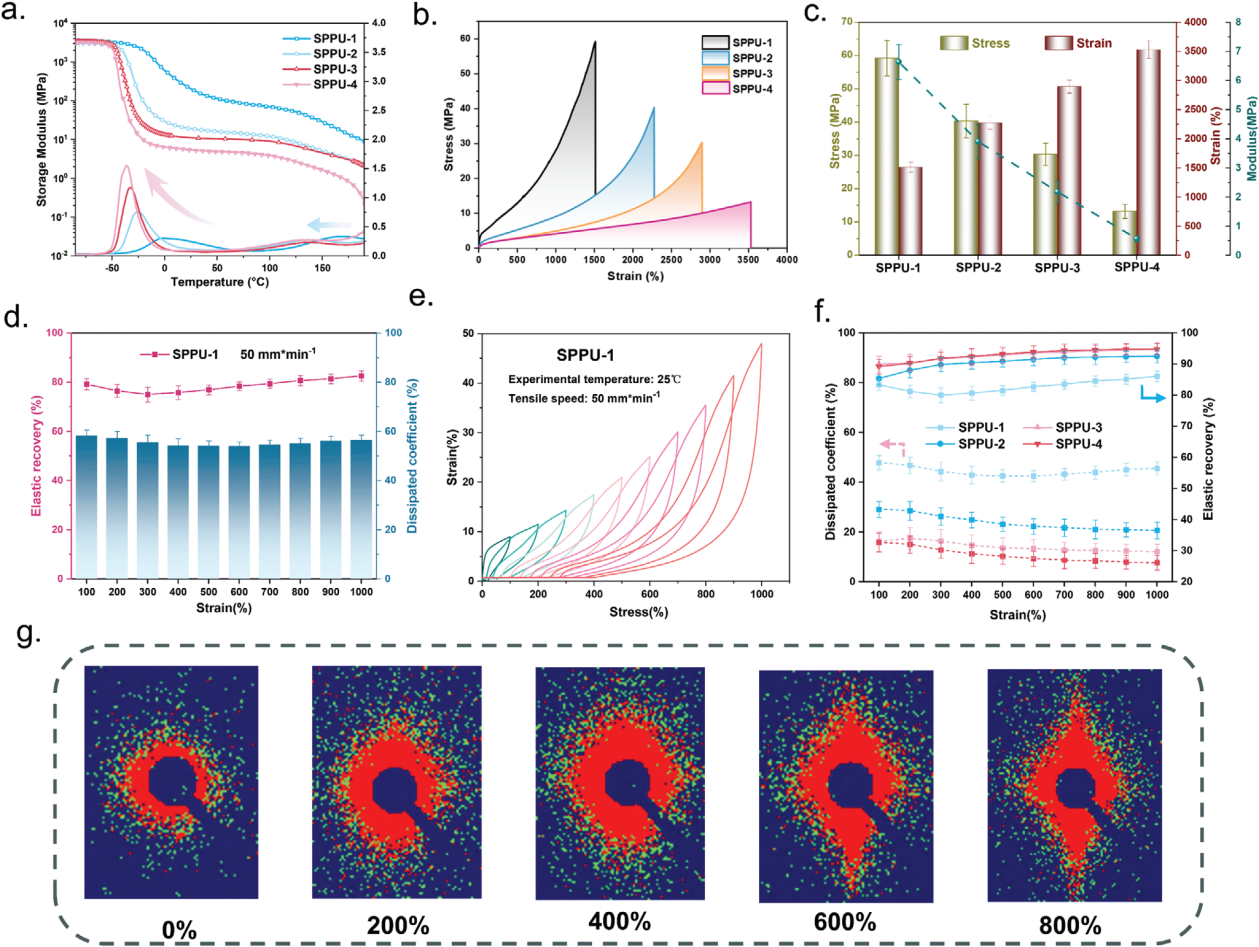
a) DMA curves for SPPUs. b) Tensile Properties Curve of SPPUs. c) Histograms of strength, elongation at break, and modulus of SPPUs. d) Strain‐increasing cyclic stretching of SPPUs, and e) elastic recovery and energy dissipation index (taking SPPU‐1 as an example). f) Comparison of elastic recovery and energy dissipation index of SPPUs. g) SPPU‐3 SAXS during stretching process.

This study provided in‐depth insights into the unique mechanical behaviors of polyurethane elastomers (SPPUs) because of their high‐density hydrogen bonds, specialized hard‐segment structures, and well‐ordered microphase‐separated configurations, endowing them with excellent flexibility and robustness. Dynamic mechanical analysis (DMA) was employed to investigate the temperature‐dependence of the energy storage modulus and *T_g_* of SPPUs. The results indicated a stable energy storage modulus at lower temperature sharply declined as temperatures approach *T*_g_, followed by a brief period of stability before a final decrease to near 0 (**Figure** **3**a). Notably, the internal dissipation angle curves exhibited two peaks corresponding to the *T*_g_ of soft and hard segments. A decrease in hard segment content leaded to a reduction in the *T*_g_ of soft segments and an increased in the peak height, signifying enhanced flexibility and damping characteristics of SPPUs. Among the samples, SPPU‐1 distinguished itself with a higher *T*_g_ for the hard segment, indicative of its dense hydrogen bonding network and the pronounced restriction effect of hydrogen bonds.^[^ ^36^ ^]^

Tensile testing on dumbbell‐shaped samples (25 mm × 4.0 mm × 1.0 mm) further elucidated the mechanical properties of SPPUs. In Figure 3b,c, SPPU‐1 showcased exceptional mechanical properties with an ultra‐high tensile strength of 59.3 MPa, an elongation at break of 1513%, and a toughness of 355 MJ m^−3^, which is superior to that of commercial elastomers.^[^ ^39^ ^]^ SPPU‐4, representing the most flexible feature, achieved a remarkable elongation at break of 3530% while maintaining a tensile strength of 13.3 MPa and toughness of 230 MJ m^−3^. As shown in Figure S8, Supporting Information, the control samples derived by altering the hard segments exhibited poorer mechanical properties (SPPU‐3_IPDA_: 14.6 MPa and 2367%; SPPU‐3_EDA_: 3.69 MPa and 2940%) compared to that of SPPU‐3 (30.2 MPa and 2890%). This can be ascribed to the fact that the benzene ring in the molecular structure of DETDA can enhance the rigidity of the molecular chain. Thus the strength of SPPUs over the six‐membered aliphatic ring structure of IPDA and the linear structure of EDA.^[^ ^40^ ^]^ Although EDA can form more ordered hydrogen bonds, the molecular chains will be too flexible to be ultra‐strong. The SPTU‐3 obtained by altering the soft segments possessed the best strength, but its molecular chain is too regular, significantly reducing its solubility properties (Tables S3 and S4, Supporting Information). Notably, all SPPUs possess higher toughness than commercial elastomers^[^ ^37^ ^]^ (200 MJ m^−3^), with SPPU‐1 having toughness comparable to the toughest spider silk (354 MJ m^−3^). As shown in Figure S9, Supporting Information, SPPU possesses low modulus of 0.5–6.7 MPa. To evaluate the energy dissipation index and elasticity of SPPUs, they were subjected to continuous cyclic stretching at constant strains (100% and 1000%) and continuous cyclic stretching at increasing strains from 100% to 1000%. SPPU‐1 exhibits the largest hysteresis loop in cyclic stretching at a constant strain of 100%, and the curve obtained from retesting at room temperature for 2 h cannot fully overlap with the initial curve (Figure S10, Supporting Information). This was attributed to the high rigidity of the molecular chain of SPPU‐1 and the poor motility of the chain segments at room temperature. In contrast, the rest of the samples, when retested after 2 h at room temperature, showed curves that almost completely overlapped with the initial ones. When the strain was increased to 1000%, the results indicated that all the samples produced large hysteresis loops only in the first cycle (Figure S11, Supporting Information). The hysteresis loops obtained from the subsequent stretching cycles remained stable, suggesting that the motions occurring in the hydrogen bonds during the initial stretching process cannot be recovered in time. Comparing the subsequent stretching cycle curves, the hysteresis loops gradually converge, indicating that the dissociation and reorganization of hydrogen bonding reached an equilibrium under this condition.^[^ ^41^ ^]^ The variations in hysteresis loops between samples suggested that the elasticity and energy dissipation capacity of SPPUs follow a certain.

The energy dissipation index (EDI) and elasticity (ER) of segmented SPPUs were quantitatively assessed through a series of cyclic stretching experiments at incrementally increasing strains, as delineated by Equations (S2) and (S3) (Supporting Information).^[^ ^42^ ^]^ As illustrated in Figure 3d–f, all SPPUs exhibit a slight decrease in EDI or reach equilibrium during strain‐increasing cyclic stretching, ascribed to incompatible bonding after partial hydrogen bonding dissociation during repeated stretching. A scrutiny reveals that the EDI of SPPU‐1 minimizes at 500%, followed by a slight increase. This phenomenon is explained by the high content and dense distribution of hydrogen bonds (Figure 2d). SPPUs presented excellent resilience, among which the ER of SPPU‐1 was higher than 80% and tended to decrease at low strains, and then gradually increased with the increment of strain. The initial decrease of ER of SPPU‐1 was attributed to the weak reorganization tendency of the broken hydrogen bonds and the poor molecular chain mobility at low strains. With the increase of strain, a large number of hydrogen bonds are broken, resulting in an increase in the efficiency of the reorganization between hydrogen bonding sites, which is macroscopically manifested as an enhancement of the resilience. Except for SPPU‐1, the ERs of all the samples reach 90% and are positively correlated with the strain, which is attributed to the excellent dissociation‐reorganization properties of the hindered hydrogen bonds. SPPUs combine exceptional resilience and energy dissipation, providing new insights into the design of novel elastomers.

Given its advantageous balance of flexibility and strength, SPPU‐3 was identified as a prime candidate for further exploration, particularly as a substrate material for alcohol recovery sensors. To validate our hypothesis on a molecular level, in situ small‐angle X‐ray scattering (SAXS) was employed to scrutinize the alterations in the hydrogen bonding‐induced phase separation structure during stretching.^[^ ^43^ ^]^ The SAXS data depicted in Figure 3g. It indicated an increase in electron density inhomogeneity in SPPU‐3 with escalating strain, signifying the orientation of hydrogen bonds and molecular chains in response to external forces. These in situ SAXS findings unequivocally confirmed the pivotal role of hydrogen bonding modifications in influencing both the elasticity and energy dissipation properties of SPPUs, thereby providing a comprehensive understanding of these SPPUs' mechanical behavior under strain.

**Figure 4:**
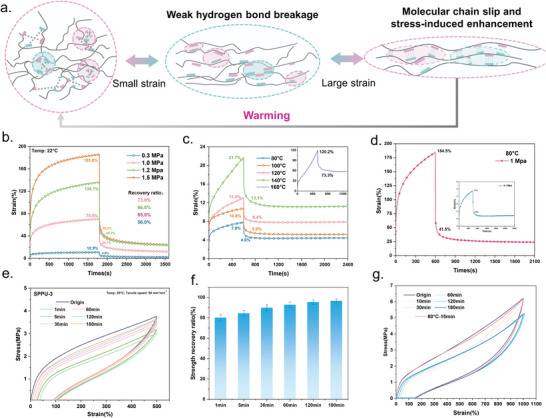
a) Schematic diagram of the changes in hydrogen bonding in SPPUs during stretching. b) Creep curve of SPPU‐3 with changing initial force. c) Variable temperature creep curve of SPPU‐3. d) Creep curves of SPPU‐3 at constant temperature (80 °C) but varying initial force. Variable response time cyclic stretching of SPPU‐3 e,f) Strain = 500%, g) Strain = 1000%.

As shown in **Figure** **4**a, hydrogen bonding in SPPUs undergoes modification in response to external forces or temperature along with the movement of the molecular chains. The hydrogen bonding in SPPUs has been investigated during strain, followed by the recovery of hydrogen bonding at different strains and the effect at different temperatures.^[^ ^37^ ^]^ As shown in Figure 4b, the pre‐applied force was varied to induce different degrees of strain in the material and held for 30 min while maintaining the temperature at 22 °C, and then the external force was withdrawn to observe the recovery ratio of SPPU‐3 under different strains. We choose 30 s after the release of the external force to calculate the recovery ratio, and found that recovery ratio is positively correlated with the strain, which is consistent with the results of Figure 3d,e. And further confirms that increasing the strain activates the more movement of stronger hydrogen bonds, which in turn improves the ER. The control samples SPPU‐3_IPDA_ showed the same trend (Figure S13, Supporting Information). Subsequently, the variable temperature creep curve of Figure 4c was obtained by changing the creep temperature while maintaining the applied external force constant. Surprisingly, even when SPPU‐3 was kept under 0.1 MPa stress for 8 min at 160 °C, the hydrogen bonding still ensured that it recovered about 40% strain after stress release, indicating that the hydrogen bonding in SPPU‐3 still existed and functioned even at 160 °C. Based on the above creep results, we further investigated the recovery ability of SPPU‐3 at different strains at high temperatures. The conditions chosen were temperature 80 °C and set stresses of 0.1 and 1.0 MPa. As shown in Figure 4d, the recovery ratio reaches 77.5% at a stress of 1.0 MPa, while the recovery ratio is only 37.2% at 0.1 MPa, more than twice as much, attributed to the fact that the initial force of 1 MPa causes SPPU‐3 to undergo a larger creep and more hydrogen bonds are activated. And this effect is much more than the molecular chain movement induced by the temperature increase. These studies demonstrate that hydrogen bonding in SPPUs is gradually activated with increasing strain. Even these hydrogen bonding remains in SPPUs at high temperatures (160 °C). The results of the elevated temperature rheology measurement further confirm this inference (Figure S14, Supporting Information). Based on the study of the effects of hydrogen bonding on the mechanical properties of SPPUs at different strains as well as temperatures, the next step is to investigate the recovery of hydrogen bonds after they are broken. In order to further evaluate the elastic recovery ability of SPPUs, we stretched the SPPUs to a fixed strain and then varied the rest time to obtain different tensile cycling curves. The strain of cyclic stretching was first fixed at 500%, and the rest time was gradually extended after each stretch from 1 min to 3 h. As shown in Figures 4e,f and S15, Supporting Information, all the samples of SPPUs could recover about 80% of their strengths after a 1 min rest. In the case of SPPU‐3, for example, the tensile strength and hysteresis loop area of SPPU‐3 gradually recovered and approached the initial values with the increase of rest time. Subsequently, the elongation was increased from 500% to 1000%, and the rest time was appropriately extended to obtain Figure 4g. Remarkably, when the strain was fixed at 1000%, SPPU‐3 exhibited a gradual recovery of mechanical properties with the extension of recovery time during the first three tensile cycles. However, after three cycles of tensile stretching, the tensile strength and toughness of SPPU‐3 decreased dramatically and could not be recovered to the initial state even if the rest time was continued to be extended to 3 h. This was attributed to the fact that the molecular chains slipped after the dissociation of the strong hydrogen bonding and the new hydrogen bonding was formed under the action of the external force to immobilize a part of the transient strain, which prevented it from being recovered to the initial state.^[^ ^44^ ^]^ Subsequently, SPPU‐3 was placed in an oven at 80 °C for 15 min, and was stretched again to reveal that its tensile strength and hysteresis loop area almost overlapped with the initial state, which was attributed to the activation of hydrogen bonding at high temperatures, which greatly enhanced the motility of the chain segments, and the molecular chains were rearranged to the optimal state (as shown in Figure 4a). Conclusively, by virtue of SPPUs' unique benzene ring‐based hydrogen bonding formed by hindered diamines, they exhibit both excellent kinematics and strength, realizing an excellent balance of elasticity and strength.

### 2.3 Alcohol Solvent Recovery and Recovery Stability of SPPUs

**Figure 5:**
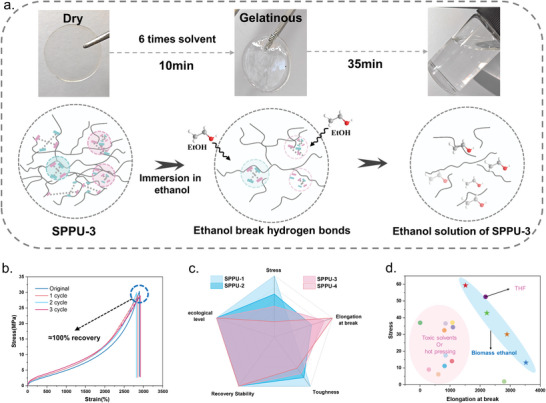
a) Schematic diagram of ethanol dissolution of SPPUs. b) Mechanical properties of SPPU‐3 before and after solvent recovery. c) Radar plot for comprehensive performance comparison of SPPUs. d) Comparison of the mechanical properties of SPPUs with the reported ones of recyclable polyurethanes.

Faced with the increasingly serious problems of white pollution and resource waste in modern society, the realization of low‐cost and high‐efficiency recycling of waste plastics has garnered widespread attention.^[^ ^45^ ^]^ Traditional thermoplastic materials can be recycled by using high temperature and pressure or a massive number of toxic solvents, which is not only energy‐consuming and expensive but also involves the leakage of organic solvents that threaten personal health. The unique molecular chain structure of SPPUs, with numerous side methyl groups in the soft segment of PPG, the high flexibility of IPDI and the double ethyl‐substituted structure of DETDA, prompts SPPUs to form hydrogen bonds with both strength and spatial resistance. This endows the molecular chain with excellent mobility and enables it to be dissolved quickly by alcohol‐based solvents. Although most linear polyurethanes can be dissolved in DMF, THF and DMAc, these solvents still suffer from the disadvantages of high toxicity, difficulty in evaporation, and high cost, making large‐scale use challenging.^[^ ^24^, ^46^, ^47^ ^]^ SPPUs, however, can be dissolved in biomass alcohol solvents (e.g., ethanol) and display a rapid dissolution rate at room temperature, effectively overcoming the aforementioned issues. Simultaneously, the dried sample of SPPUs can recover its mechanical performance and transparency after soaking in water for 8 (Figure S16, Supporting Information). When the SPPUs were immersed in ethanol, the ethanol molecules began to break the hydrogen bonds formed within the SPPUs molecules,^[^ ^48^, ^49^ ^]^ rapidly dissolving the SPPUs to form an ethanol solution at room temperature (**Figure** **5**a). Subsequently, the ethanol is removed to obtain the recovered SPPU film. As shown in Figures 5b and S17, Supporting Information, the recovered SPPUs can maintain almost identical mechanical properties to the initial SPPUs, achieving nearly 100% recovery of mechanical properties. Additionally, the use of a biomass solvent (ethanol) for recycling SPPUs is both biologically eco‐friendly and easily removable, providing a significant advantage over pre‐existing recyclable materials. The comprehensive comparisons among different SPPUs were listed in Figure 5c, including recycling stability, eco‐friendliness, and mechanical properties, resulting in radar charts. Notably, all samples exhibited excellent recycling stability and eco‐friendly properties, with toughness significantly superior to that of commercial elastomers, achieving an optimal balance between green recycling and mechanical properties. Further comparing the SPPUs with existing recyclable polyurethanes (Figure 5d), most existing recyclable polyurethanes are recycled using solvents such as DMF, DMAc, and THF or through hot pressing, which is not only environmentally unfriendly and wasteful but also results in generally low‐strength materials.^[^ ^24^, ^46^, ^47^, ^50^, ^51^, ^52^, ^53^, ^54^, ^55^, ^56^, ^57^, ^58^ ^]^ However, the SPPUs can overcome these challenges by enabling room temperature recycling with biomass solvents while maintaining high strength (up to about 60 MPa) and high elongation at break. Therefore, the successful synthesis of SPPUs contributes to the further exploration of realizing the high strength and toughness in polyurethane and green solvent recovery.

### 2.4 Recyclable Capacitive Sensors

**Figure 6:**
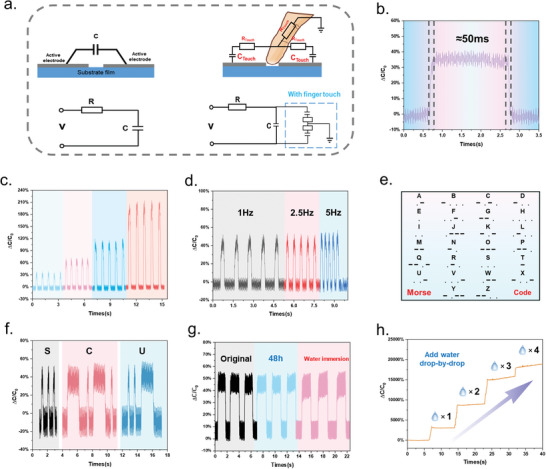
a) Schematic diagram of the sensing principle of capacitive sensors. b) The response and recovery time of PCBS upon touch. c) Capacitive response as a function of the number of fingers. d) Capacitor response as a function of touch frequency. e) Table of Morse code representing each letter. f) Presentation of PCBS to convert touch signals into electrical signals (“S,” “C,” and “U”). g) Stability of PCBS in storage (48 h) and water resistance (5 min immersion in water). (h) Capacitive response as a function of the number of water drops.

Capitalizing on the excellent alcohol solubility of SPPU‐3, the paste of SPU‐3_1/3Ni_ was selected as the matrix to fabricate a comb‐shaped capacitance‐based sensor through screen printing. This sensor named printable capacitive‐based sensors (PCBS) can be printed onto PET film, paper and SPPUs. The cross‐sectional and surface micro‐morphology of PCBS printed on different substrate materials were characterized using SEM and EDS (Figures S24–S26, Supporting Information). PCBS consisted of multiple interpenetrating comb electrodes, utilizing the edge effect to transform external variation into electrical signals. First, the cyclic pressure test and continuous pressure test data for this capacitive sensor is shown in Figure S27, Supporting Information, indicating that this PCBS possess sensitive stimulus responsiveness as and cyclic stability. **Figure** **6**a illustrated the principle of fringe field capacitive sensing. Then, we varied the number of fingers in contact with the PCBS and the contact time to investigate the change in capacitance. As shown in Figure 6c,d, the PCBS can accurately distinguish the breakup index, as more fingers (i.e., a larger effective contact area) produce higher capacitance changes. Similarly, the capacitance signal persisted with constant intensity when varying the number of fingers (Figure S28, Supporting Information). Based on this feature, PCBS enabled message encryption and delivery. In Morse code (Figure 6e), the click and long press on PCBS corresponded to “dot” and “cross,” respectively, mimicking a radio station to realize the secure transmission information. Figure 6f showed the information converted by PCBS, displaying “S,” “C,” and “U” for enabling the precise data transmission. This allows for the design of customized signal conversion tables for personalized information encryption. Notably, PCBS also possessed an extremely fast response time (about 50 ms), which is significantly better than all capacitive sensors reported thus far (Figure 6b). This characteristic was attributed to the remarkably conductivity of Ni powder‐formed conductive pathways, largely reducing signal attenuation and energy loss. In Figure 6d, the capacitance changes of PCBS at different touch frequencies confirmed that the PCBS can maintain a high responsiveness along with a particularly high resolution. Hence, it can operate at high frequencies and maintain a stable signal output. Durability and stability are also important evaluation indexes for flexible sensors. Figure 6g demonstrated that PCBS can maintain the same signal output as the initial one even after 48 h of storage or immersion in water, exhibiting the excellent stability of PCBS in use. Based on its water resistance, PCBS can also be used for early warning of water leakage. As shown in Figure 6h, water droplets generated a huge signal for the PCBS (much higher than that of a finger). Increasing the number of water droplets presents a corresponding stepped change in signal, which is attributed to the extremely high dielectric constant of water. As illustrated in Figure S29, Supporting Information, by leveraging the difference in dielectric constants of different objects and the variation in contact area with the PCBS, the sensor can also achieve object identification based on the different signals generated by different objects. Noticeably, the capability of PCBS to realize functions such as information encryption and transmission and object identification paves the way for its future application in wearable human‐computer interaction. Additionally, SPPU‐3_1/3Ni_ ink can be printed on PET film, SPPU3 film as well as paper, exhibiting high‐sensitive, well‐resolution sensing performance (Figures S30 and S31, Supporting Information).

**Figure 7:**
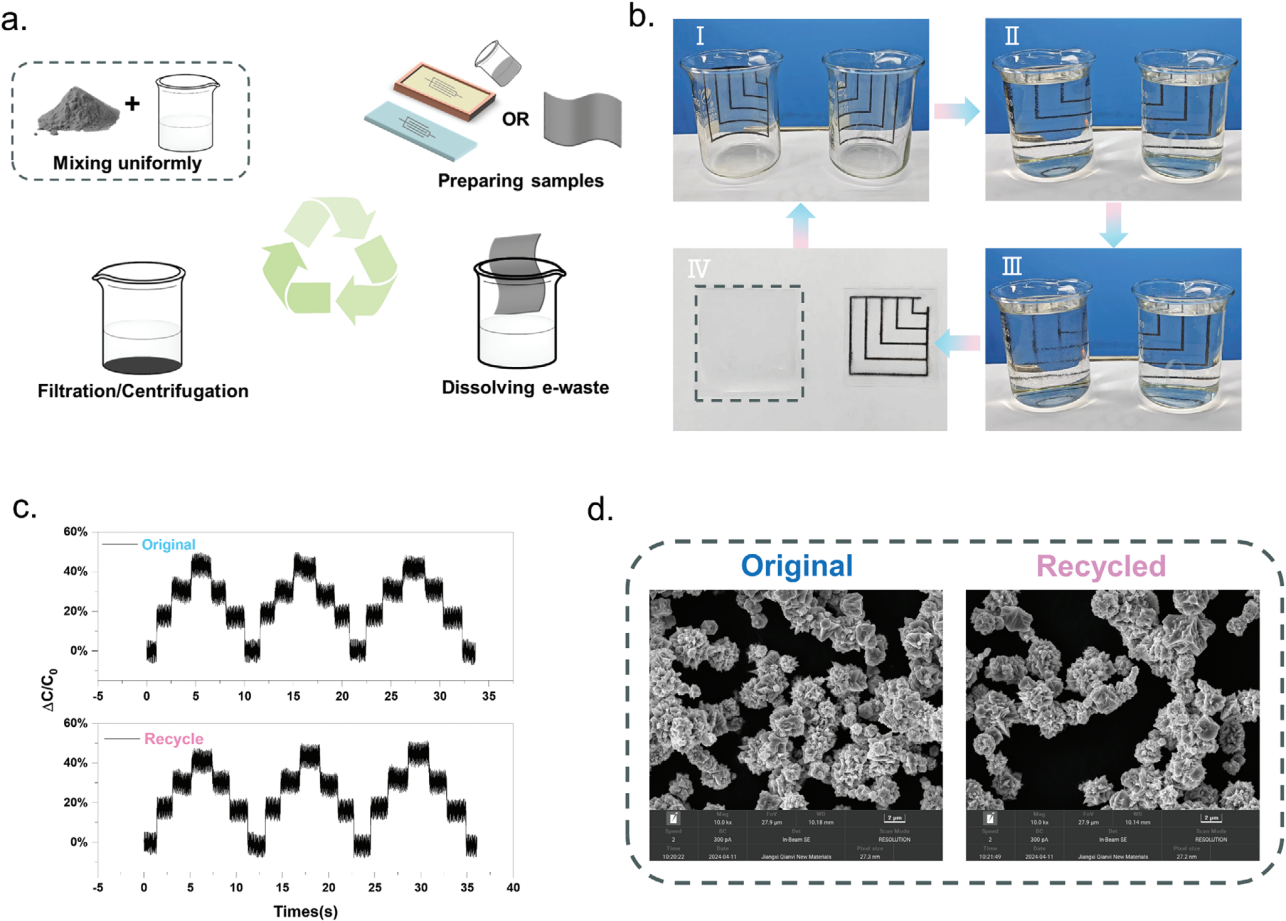
a) Schematic diagram of the recovery of PCBS. b) Recovery of PCBS in ethanol solvent (left) and stability in water (right). c) Comparison of PCBS sensing performance before and after recycling. d) SEM images of Ni powder before and after recycling.

Recently, the ever‐increasing e‐waste became a huge source of environmental pollution. For this reason, transient circuits and the recyclable electronics development have become a mainstream trend.^[^ ^59^, ^60^ ^]^ Although water‐soluble electronics as reported in some studies, the ubiquitous water in our lives causes these electronic products to be extremely limited in their applications and less reliable in their use. With the superior stability of alcohol‐soluble transient circuits over water‐soluble ones, alcohol‐soluble transient circuits show a tendency to become the next generation of recyclable sensors. **Figure** **7**a shows a schematic diagram of the recovery of PCBS. Digital pictures of the recovery process are presented in Figure 7b, where the circuits were immersed in ethanol (left) and water (right), respectively. The circuit immersed in ethanol had visible dissolution after 10s, and after 1 min the circuit was already mostly dissolved, and 3 min the circuit disappeared into obscurity. However, the circuit immersed in water was taken out and dried after 5 min with the circuit still maintaining its pathway, indicating that the circuit based on SPPU‐3_1/xNi_ exhibits excellent water resistance and alcohol solubility. As currently reported, the recovery of conventional conductor materials remains challenging, attributed to constituents routinely being dissolved or dispersed in the matrix solution after dissolution. In contrast, due to the high density of Ni powder and its insolubility in any organic solvent, the Ni powder and the ethanol solution of SPPU can be initially separated by magnetic attraction, and then completely separated and recovered using high‐speed centrifugation, with a recovery efficiency of 99.6% for the Ni powder. Moreover, the recovered Ni powder maintained the same morphology as the initial one (Figure 7d). In addition, the recovered SPPU‐3 also possessed similar mechanical properties as the initial one (Figure S32, Supporting Information). As shown in Figure 7c, the SPPU‐3_1/3Ni_‐based PCBS obtained after mixing them again remained consistent with the pre‐recycled properties, demonstrating the efficient recycling of SPPU‐3_1/3Ni_’s excellent mechanical properties and sensing performance. Concluding, the SPPU‐3_1/3Ni_ films possessed excellent flexibility, tensile properties and strength. In addition, the recyclable and separated raw material, PCBS prepared based on it features abundant functions, such as information encryption for transmission and object recognition, exhibiting great potential for development in the field of human‐computer interaction. Notably, altering the Ni powder to carbon black, we prepared an alcohol‐soluble transient electronic device, which can be used for preparing other conductive materials (Figures S34–S36, Supporting Information and Video S1).

## 3 Conclusions

Summarily, Conclusively, we have developed a simple synthesis method using commercially available raw materials to synthesize highly transparent, high‐strength, and excellent‐toughness polyurethane‐polyurea elastomers (SPPUs). The unique benzene ring group of hindered diamines (DETDA), IPDI, and the side methyl group of PPGs in SPPUs cooperate to form disordered‐hydrogen bonds, enabling SPPUs to be readily solubilized in a renewable ethanol. Compared to conventional recyclable linear polyurethanes, SPPUs offer significant advantages in environmental friendliness, alongside the high strength of ≈60 MPa and toughness higher than 350 MJ m^−2^. Utilizing this characteristic, the devised recyclable and prin capacitive‐based sensors (PCBS) have high response time only 50 ms, high resolution at a frequency of 5 Hz, and flexibility via screen printing. This PCBS can recognize the number and type of objects, enable encrypted transmission of information, and monitor water vapor. Intriguingly, waste SPPU‐31/xNi can be substantially separated and fully recovered by centrifugation, and the recovery efficiency of Ni powder and SPPUs was close to 100%. In particular, the PCBS exhibits consistent sensing capabilities before and after recycling, possessing excellent recycling stability, significantly advancing the development of transient, recyclable circuits. Our preparation method is compatible with various conductive fillers (e.g., liquid metals, AgNWs, CNTs), recyclable polymers (e.g., PCL, PLA), and microelectronic devices (e.g., humidity detectors, gas detectors). It is potential to design transient, recyclable electronics with diverse functionalities, facilitating the development of wearable smart devices. The synthesis of alcohol‐soluble recyclable SPPUs and the fabrication of PCBS presented here will address the issues of conductive filler leakage, matrix material susceptibility to water, and contamination in the recycling process, thereby enabling the realization of transient circuits and sensor devices for a wide range of applications.

## 4 Experimental Section

Experimental details are provided in the Supporting Information.

## Conflict of Interest

The authors declare no conflict of interest.

## Supporting information



Supporting Information

Supplemental Video 1

## Data Availability

Research data are not shared.
