# Supplementary material for: Enabling High Strength and Toughness Polyurethane through Disordered‐Hydrogen Bonds for Printable, Recyclable, Ultra‐Fast Responsive Capacitive Sensors
Source: Adv Sci (Weinh). 2024 Oct 14;11(45):2405941. doi: 10.1002/advs.202405941 (PMC11615776; doi:10.1002/advs.202405941)
Supplement: Supplementary file 1 — Supporting Information [file ADVS-11-2405941-s001.docx]

**Supporting Information**

**Enabling High strength and toughness Polyurethane Through Disordered-Hydrogen Bonds for Printable, Recyclable, Ultra-Fast Responsive Capacitive Sensors**

Xingbao Chen, Shiwei Zhao, Anqian Yuan, Silong Chen, Yansheng Liao, Yuan Lei, Xiaowei Fu, Jingxin Lei*, Liang Jiang*

*State Key Laboratory of Polymer Materials Engineering,* *Polymer Research Institute of Sichuan University, Chengdu 610065, China.*

* Corresponding authors.

E-mail addresses: [jxlei@scu.edu.cn](mailto:jxlei@scu.edu.cn) (J. Lei); [liangjiang@scu.edu.cn](mailto:liangjiang@scu.edu.cn) (L. Jiang).

**CONTENTS:**

**1. Experimental Section**

**2. Characterization**

**3. Tables**

**4. Figures**

**5. Videos**

**1. Experimental Section**

**1.1 Materials**

Polypropylene glycol (PPG, M_n_ = 1000, 2000, 3000 and 4000 g mol^-1^) were purchased from ShandongBluestar Dongda Chemical Co. Ltd (China). Polytetramethylene ether glycol (PTMEG, M_n_ = 2900g mol^-1^) were purchased from Sigma-Aldrich (America). Poly (ethylene glycol) (PEG, M_n_ = 3000g mol^-1^), Ethylenediamine (EDA), Ethyl Alcohol (EA) and Dibutyltin dilaurate (DBTDL, 95%) were purchased from Chengdu Kelong Chemical Reagent Company (China). Isophorone diisocyanate (IPDI), Diethyl methyl benzene diamine (DETDA) and Isophorondiamine (IPDA) was purchased from Adamas (China). Ni powder purchased from Brofos Nanotechnology Co. Ltd. All reagents and solvents were not further purified without specific instructions. Ethyl acetate (EA) was analytical grade and purchased from Kelong Chemical Reagent Co. Ltd (Chengdu, China), dehydrating with 5 Å molecular sieve for 15 days prior to use.

**1.2 Synthesis of the PUs**

The typical synthetic routes for the SPPU-1, 2, 3 and 4, SPPU-3_IPDA_, SPPU- 3_EDA_, SPEU-3 and SPTU-3 polymers are described in detail as follows. The preparation process of **SPPUs** is simple and consists of a two-step reaction, the preparation of prepolymer and chain expansion reaction. Here we take SPPU-3 as an example, and the preparation conditions of other materials are consistent with it. A three-necked round-bottomed flask equipped with a mechanical stirrer was charged with PPG (M_n_=3000 g mol^-1^, 10 g, 3.33 mmol), which was then heated and stirred under vacuum at 105°C for 60 min to remove moisture. Subsequently, the reaction system was cooled down to 60°C and IPDI (1.51 g, 6.73 mmol) was added and reacted at 60°C for 1 h. After reacting at 60°C for 1 h, a drop of DBTDL (0.02 g) was added to the system and the temperature was raised to 80°C to continue the reaction for 3 h. The reaction endpoint was determined using an NCO value titrator (CT-1Plus Automatic Potentiometric Titrator, China). Followed by cooling to room temperature and adding 20 g of dewatered EA and mixing homogeneously, DETDA (0.54 g, 3.03 mmol), a chain extender dissolved in 5 g of dewatered EA, was added, and after mixing uniformly, the solution was poured into a tetrafluoro mold. It was left at room temperature for 4 h, then in a blast drying oven at 60°C for 12 h and at 80°C for 24 h. Finally, it was left in a vacuum drying oven at 60°C for 6 h to remove all solvents. SPPU-3_IPDA_ and SPPU- 3_EDA_ due to the fast chain expansion reaction, the amount of solvent added is two times of the above. SPEU-3 is crystallized at low temperature, so the solvent was chosen to be dewatered DMF and placed in an oven at 80 ℃ for the subsequent reaction after mixing well.

The reactive endpoint of the prepolymer preparation stage was determined by the NCO value obtained by titration. The molecular weights (*M_w_*) of the SPPU-1, 2, 3 and 4 polymers were measured to be ca. 120 000 g mol^-1^, 60000 g mol^-1^, 170 000 g mol^-1^ and 80000 g mol^-1^, respectively. SPPU-3_IPDA_, SPPU-3_EDA_ polymers were measured to be ca. 1.8×10^5^ g mol^-1^, 1.2×10^5^ g mol^-1^, respectively. SPEU-3, SPTU-3 polymers were measured to be ca 4.0×10^6^ g mol^-1^ and 5.0×10^4^ g mol^-1^.

**1.3 Preparation of the SPPU-3_1/xNi_**

In the case of SPPU-3_1/3Ni_, 3 g of SPPU-3 was dissolved in 15 g of ethanol, followed by adding 9 g of Ni powder, which was mixed under mechanical stirring and simultaneously heated to 50°C to remove the solvent. When the viscosity had risen, the solution was poured into a tetrafluoro mold after it had cooled down to room temperature with stirring. The thickness of the film obtained was 0.3 mm.

**1.4 Preparation of the printable capacitive sensors (PCBS)**

According to the method in **1.3**, a paste of SPPU-3_1/3Ni_ was formulated. After that, it was printed on PET film, SPPU films and paper using screen printing. Stencil printing is conducted with a 20 cm × 15 cm hardwood screen printer using emulsion screens and polyester screen-printing mesh (80 mesh, 198 microns per inch of threat, opening). A polyurethane squeegee (at a 45° angle to the mesh) was used to brush the ink onto the substrate. The printed SPU-3_1/3Ni_ is then dried at room temperature for 2 h and then at 60°C for 1 hours. The detailed dimensions of the PCBS are shown in the Figure S33.

**1.5 Recycling of SPPUs**

In case of SPUU-3, for example, 5 g of SPUU-3 was immersed in a 50 ml beaker containing 25 g of ethanol and stirred magnetically at room temperature. After waiting for complete dissolution (about 1.5 h at room temperature), the solution was poured into a tetrafluoro mold and the solvent was slowly removed at room temperature, the viscosity was increased and placed in an oven at 60°C to further remove the solvent, and finally all the solvent was removed in a vacuum drying oven at 60°C.

**1.6 Recycling and separation of SPPU-3_1/xNi_ and PCBS**

5 g of SPPU-3_1/xNi_ or CBS was dissolved with 15 g of ethanol, followed by separation of Ni powder in SPPU-3 solution by high-speed centrifugation. The separated Ni powder was rinsed repeatedly with ethanol, after which the solvent was removed and weighed. The SPPU-3 solution was similarly desolventized to obtain the SPPU-3 film. The obtained SPPU-3 film can be dissolved again with ethanol and following the preparation method of **1.3** or **1.4**, where SPPU-3_1/xNi_ or PCBS can be prepared. The PCBS recovery process is referenced above.

**1.7 Water Resistance Test for SPPUs**

Taking SPPU-3 as an example, it was soaked in water for 1 h, 3 h and 8 h respectively, and then removed and wiped dry for direct testing. In particular, the soaked 8 h of SPPU-3 was removed and dried to conduct the test for comparison. The dimensions of the test samples were consistent with the tensile samples.

**1.8 Preparation of SPPU-3_10%CB_ and applications**

SPPU-3 was dissolved in 5x ethanol, followed by the addition of CB dispersed by sonication with ethanol and stirred well. Subsequently, the temperature was warmed up to 85°C to continue stirring for 30 min to promote ethanol volatilization, and finally poured into the mold.

The SPPU-3_10%CB_ serves as a transient and recyclable wire to assemble a simple transient circuit with a power supply, copper wire, and a small light bulb. The power supply is four 1.5-volt dry cell batteries.

**1.9 PCBS compression testing**

The method used in our testing was to place a square PE foam piece with dimensions of 20 mm × 20 mm × 20 mm on top of the capacitance sensor and compress it. The instrument used was a universal tensile tester, model INSTRON 5966.

**2. Characterization**

**2.1 General characterization**

Fourier transform infrared spectroscopy (FTIR) was measured by utilizing an infrared spectrophotometer (Nicolet-560, Nicolet Corporation, USA) in the wave number range of 400-4000 cm^-1^ with a resolution setting of 4 cm^-1^. Light transmission was measured by an ultraviolet-visible photometer (UV3600, Shimadzu, Japan) in the wavelength range of 400-800 cm^-1^ with the thin sample thickness of 1 mm. The X-ray diffraction (XRD) patterns were recorded by an X-ray diffractometer (X’Pert Pro MPD, The Netherlands) with Cu Kα at 35 kV and 30 mA with 2*θ* ranges of scans from 5 to 90° at a scanning rate of 5 ° min^-1^. Molecular weights of the polymers were measured using gel permeation chromatography (GPC, HLC-8320, Japan) with N,N-Dimethylformamide (DMF) as the mobile phase at a flow rate of 1 mL min^-1^. The sample concentration was approximately 2-3 mg mL^-1^ with an injection volume of 60 μL. The hydrogen bonding as a function of temperature of the samples is supervised utilizing a variational temperature Fourier transform infrared spectrometer (In-suit FTIR) range from 20 to 200 ℃ with a detection interval of 10 ℃ at heating rate of 5 ℃ min^-1^. Infrared spectrophotometer (Nicolet-560, Nicolet Corporation, USA) was utilized in the wave number range of 400-4000 cm^-1^ with a resolution setting of 4 cm^-1^, and the hot plate is used to control the temperature. The thermodynamic behavior was characterized utilizing a dynamic mechanical analyzer (DMA Q850, USA), including modulus and creep-recovery behaviors. The mechanical properties before and after recycling were characterized by an Instron-4302 Instron machine (USA) by using a 1 KN transducer at stretched rate of 50 mm min^-1^ at room temperature. The cyclic tensile test conditions were programmed for a tensile speed of 50 mm min^-1^. The sample strips used for tensile testing are dumbbell-shaped, with the stressed portion being 4 mm wide and 1 mm thick. The tensile conditions for sensing performance are consistent with those described in the previous paragraph. The evaluation of rheological behavior was investigated by a Haake MARS III (Thermo Electron GmbH, Germany) rotational rheometer with an oscillatory mode. The size of all the samples was 25 mm in diameter and ~1mm in thickness. The dynamic frequency range was set from 0.1 to 100 Hz at a range of temperatures. The thermal properties were tested by differential scanning calorimetry (DSC 204F1, Germany), in the temperature range of -70-180 ℃. The samples were placed in a N_2_ atmosphere and heated from -70 to 180°C at a rate of 10°C min^−1^. Thermogravimetric analysis (TGA, SDTQ600, USA) was used to analyze the thermal degradation behavior of SPPUs within the temperature range from 30 to 600°C, at the heating rate of 10°C min^-1^ in a nitrogen atmosphere. Surface morphology and element distribution of SPPU-3_1/xNi_ and PCBS were obtained by Navo NanoSEM450 scanning electron microscope (SEM, Apreo S HiVoc, ThermoFisher Scientific Co., UK) and energy dispersive spectroscopy accessories (EDS). Sensing performance is measured with the Digital Benchtop Multimeter (DMM6500, KEITHLEY). AFM measurements were performed on a Bruker Multimode 8 with a Nanoscope V controller in tapping-mode. The samples used for AFM testing were all frozen and sliced (Freeze ultra-thin slicers, German, Leica EM UC7/FC7).

**2.2 Small-angle X-ray scattering (SAXS) measurements**

SAXS measurements were carried out on a modified-Xeuss system with a multilayer focused Cu Kα X-ray source (GeniX3D Cu ULD, Xenocs SA, France, λ= 0.154 nm). The sample-to-detector distance is 1035 mm. One SAXS image was recorded within the exposure time of 15 min by a Pilatus 100 K detector with 487 × 195 pixels (resolution: 172 × 172 μm^2^). The one-dimensional SAXS curves were integrated by averaging azimuthally 360° of 2D SAXS patterns via the Fit2D software. The periodicity (L) was calculated by the Bragg’s law:

$L=\frac{2\pi}{q_{max}}$ (1)

where *q_max_* corresponds to the peak position of one-dimensional SAXS curve.

**2.3 Cyclic Tensile Test**

Constant strain cyclic stretching (100% and 1000%): Tensile rate of 50 mm min^-1^, 10 cycles.

Increasing strain cyclic stretching (100%-1000% at 100% intervals): Stretching rate 50 mm min^-1^.

Constant strain, variable rest time cyclic stretching (100%-500%): rest time 1min-5 min-30 min-60 min-120 min-180 min. Stretching rate 50 mm min^-1^.

**2.4 Energy Dissipation Index and Elasticity Coefficient**

Based on the results of increasing strain cyclic stretching (100%-1000% at 100% intervals), the Energy Dissipation Index and Elasticity Coefficient can be calculated as follows:

$Energy Dissipation Index=100\%\left( \left( \int_{loading} \text{σ}ⅆ\varepsilon-\int_{unloading} \text{σ}ⅆ\varepsilon\right)/{\int_{loading} \text{σ}ⅆ\varepsilon} \right)$ (2)

where σ and ε represent the stress and strain, respectively.

$ER=100\%\left( \varepsilon_{max}-\varepsilon_{b} \right)/\left( \varepsilon_{max}-\varepsilon_{a} \right)$ (3)

where ε_max_ is the maximum strain of the loading curve in a given cycle, ε_b_ is the strain at zero stress of the unloading curve in a given cycle, and ε_a_ is the strain at zero stress of the loading curve in a given cycle.

**2.5 Sensing Performance Characterization**

SPPU-3_1/xNi_, which was tailored to a dumbbell type (same as above), was stretched using a uniaxial stretching device (in **2.1**) at a speed of 50 mm min^-1^. At the same time, a conductive copper tape was used to connect the SPPU-3_1/xNi_ to a Digital Benchtop Multimeter set to the two-wire resistance mode, and the change in resistance of the SPPU-3_1/xNi_ was recorded during the stretching process.

Digital Benchtop Multimeter set to capacitance mode was used to test the capacitance change of the PCBS in use.

**3. Tables**

**Table S1.** Feeding compositions for synthesis of SPPUs.

| **Samples** | **PPG (g)** | **IPDI (g)** | **DETDA (g)** | **EA(g)** | **HS content* (%)** |
| --- | --- | --- | --- | --- | --- |
| SPPU-1 | 10 | 4.44 | 1.78 | 15 | 38.35 |
| SPPU-2 | 10 | 2.22 | 0.89 |  | 23.72 |
| SPPU-3 | 10 | 1.48 | 0.59 |  | 17.15 |
| SPPU-4 | 10 | 1.11 | 0.44 |  | 13.42 |

*HS content: Hard segment content (%).

**Table S2.** Feeding compositions for synthesis of control samples.

| **Samples** | **PPG (g)** | **IPDI (g)** | **chain extender(g)** | **Solvent (g)** |
| --- | --- | --- | --- | --- |
| SPPU-3_IPDA_ | 10 | 1.48 | IPDA-0.56 | EA-15g |
| SPPU-3_EDA_ |  |  | EDA-0.2 | EA-15g |
| SPEU-3 |  |  | DETDA-0.59 | DMF-15g |
| SPTU-3 |  |  | DETDA-0.59 | EA- 15g |

**Table S3.** Glass transition temperatures (*T_g_*), Tensile strength (σ) and elongation at break (ε) of SPPUs.

| Samples | *T_g_* (°C)  (Soft / hard section) | σ (MPa) | ε (%) |
| --- | --- | --- | --- |
| SPPU-1 | -2.73 / 166.5 | 59.2±4.3 | 1512.5±86.3 |
| SPPU-2 | -25.7 / 138.6 | 40.4±3.57 | 2275.3±103.5 |
| SPPU-3 | -32.6 / 136.5 | 30.3±3.34 | 2898.1±110.2 |
| SPPU-4 | -36.3 / 130.8 | 13.2±2.12 | 3530.3±150.3 |

**Table S4.** Tensile strength (σ), elongation at break (ε) of control samples.

| Samples | σ (MPa) | ε (%) |
| --- | --- | --- |
| SPPU-3_IPDA_ | 14.7±1.4 | 2369.2±67.2 |
| SPPU-3_EDA_ | 6.12±1.1 | 3024.2±121.5 |
| SPEU-3 | 34.8±2.2 | 2318.6±104.8 |
| SPTU-3 | 49.2±2.1 | 2017.5±94.6 |

**Table S5.** Molecular weights of SPPUs and control samples.

| Samples | *M_w_* | *RI* |
| --- | --- | --- |
| SPPU-1 | 122750 | 7.2 |
| SPPU-2 | 59956 | 4.8 |
| SPPU-3 | 166887 | 5.2 |
| SPPU-4 | 77754 | 5.6 |
| SPPU-3_IPDA_ | 168673 | 3.4 |
| SPPU-3_EDA_ | 118662 | 4.1 |
| SPEU-3 | 404368 | 5.7 |
| SPTU-3 | 53735 | 1.6 |

**Table S6.** Comparison with some existing sensors.

|  | **Conductive components** | **Recyclability** | **Eco-friendliness** | **Response time** |
| --- | --- | --- | --- | --- |
| **This work** | Ni power | **√** | **√** | 50ms |
| [1] | Liquid metals | **√** | **√** | 100ms |
| [2] | GO- LM | ○ | ○ | 440ms |
| [3] | ILs | **√** | **√** | 400ms |
| [4] | MXene | **×** | **×** | 15-30ms |
| [5] | ILs | **√** | ○ | 300-400ms |
| [6] | ILs | **√** | ○ | 80-100ms |
| [7] | Hydrogel | **×** | **×** | 120ms |
| [8] | Carbon | **×** | **×** | 66ms |

**√**: possession.

**×**: No such function.

○: Partially functioning (e.g. reprocessed but not separated for recycling).

**4. Figures**


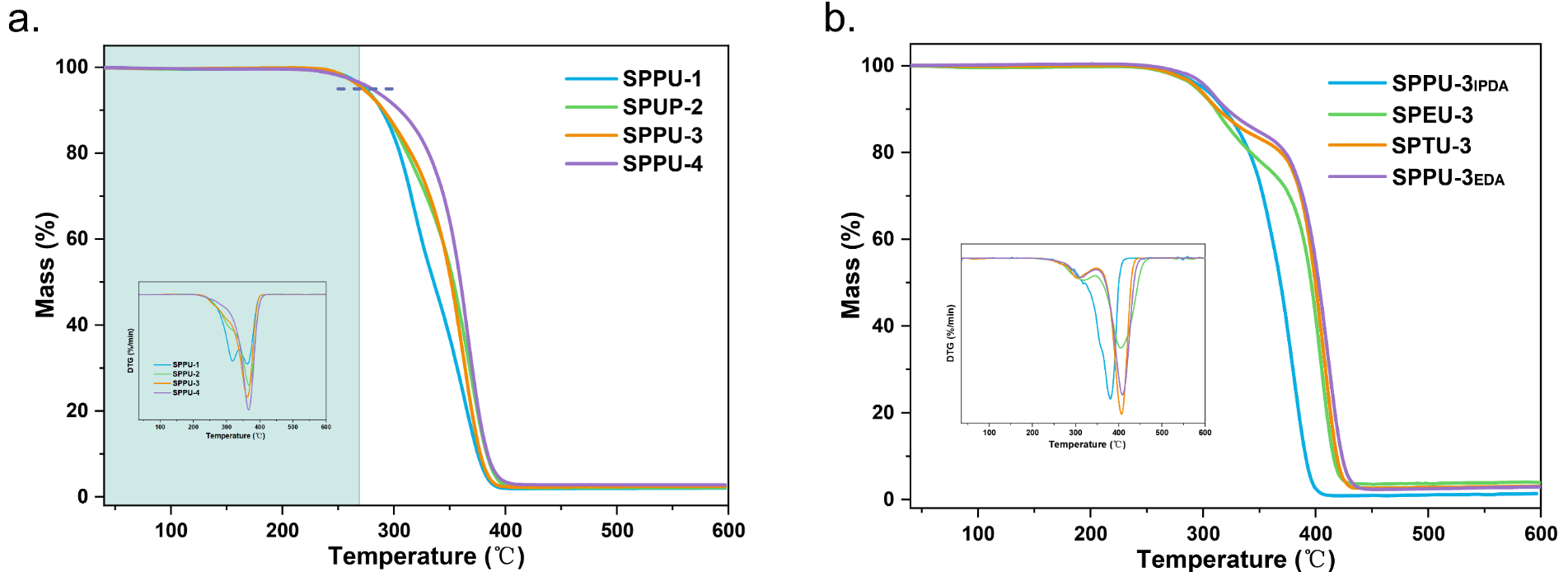


Figure S1. Thermogravimetric curve of (a) SPPUs and (b) control samples.


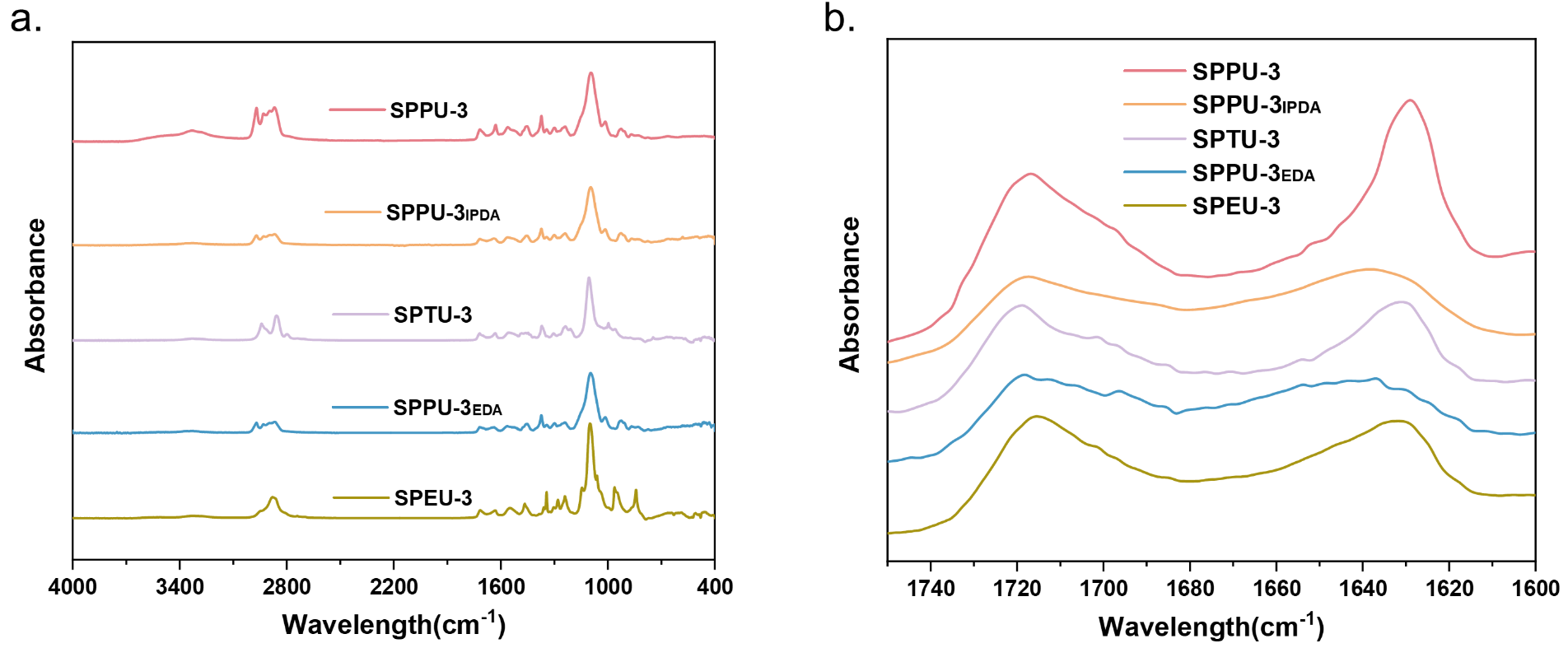


Figure S2. (a) FT-IR spectra of the SPPU-3 and its control samples. (b) Comparison of localization (1750cm^-1^-1600 cm^-1^).


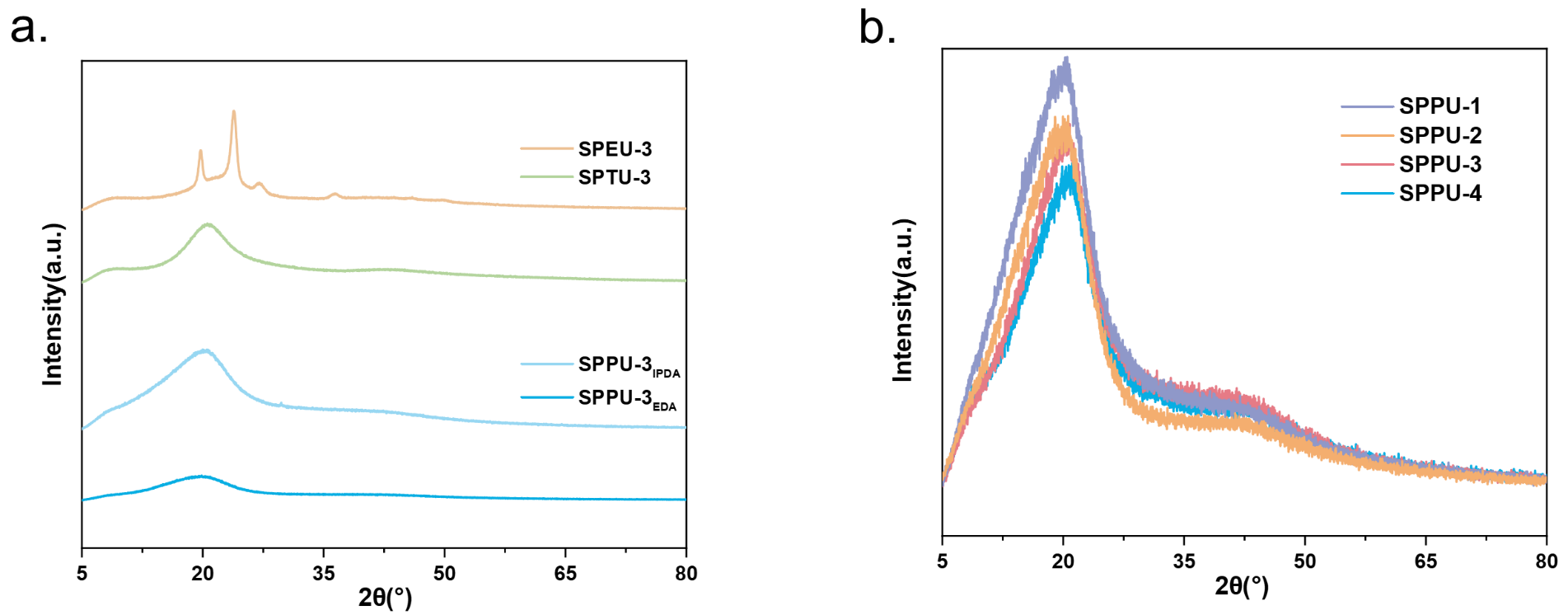


Figure S3. XRD spectra of (a) control samples and (b) SPPUs.


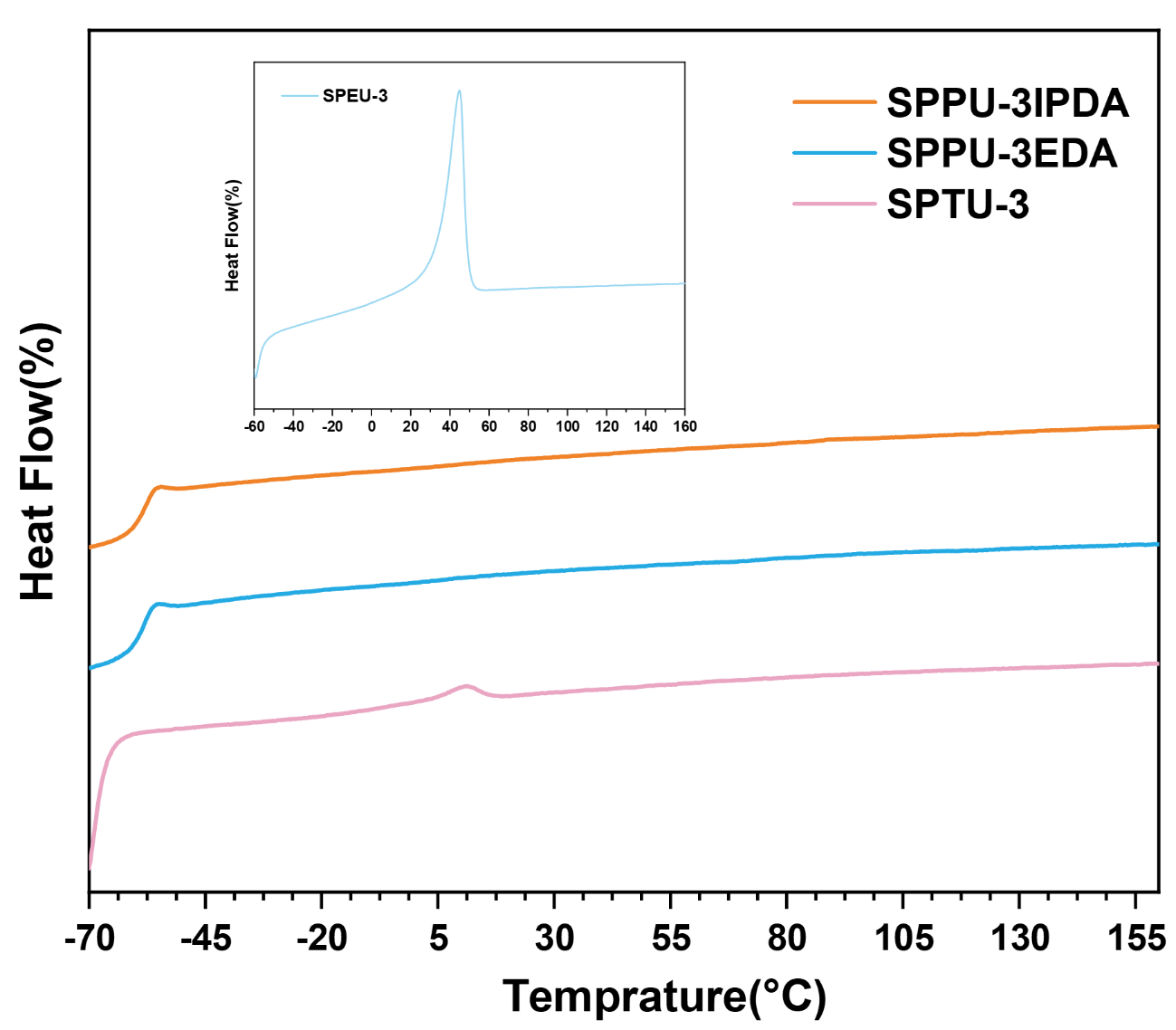


Figure S4. DSC spectra of control samples.


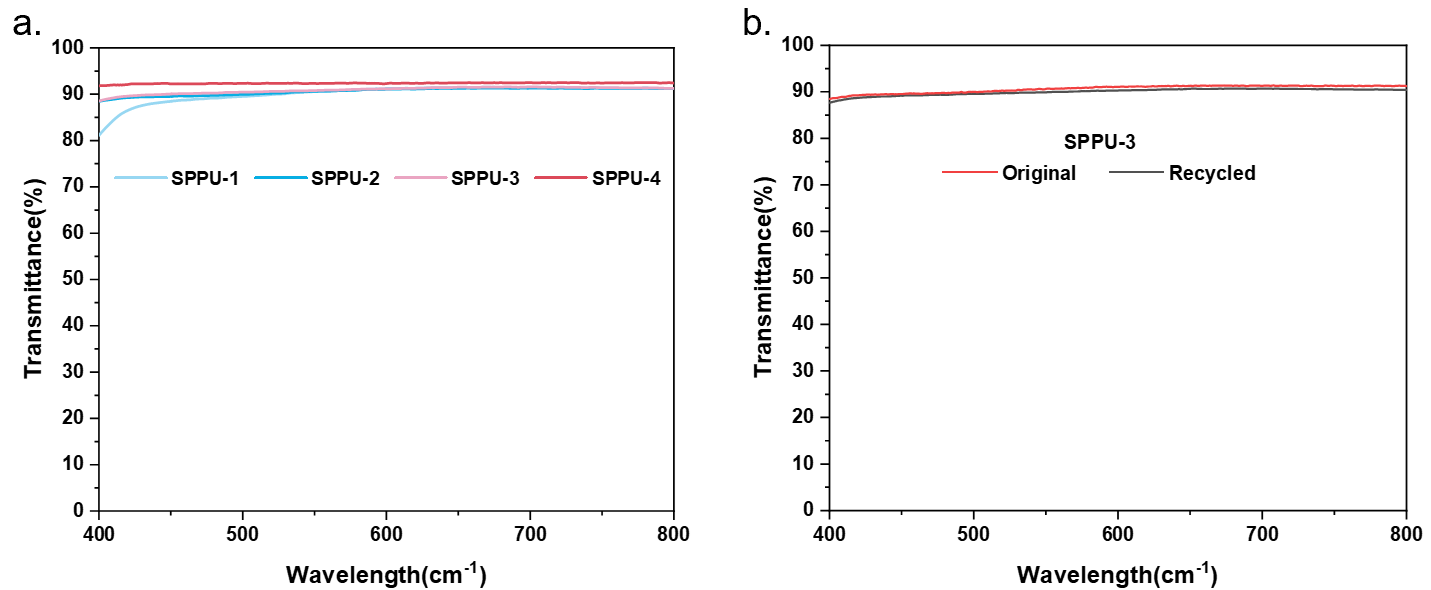


Figure S5. (a) Transmittance of SPPUs, (b) Original transmittance of SPPU-3 and recovered ones.

Because the recovery process for SPPUs involves only room temperature dissolution and solvent evaporation at relatively low temperatures (60°C), their molecular structure remains stable.


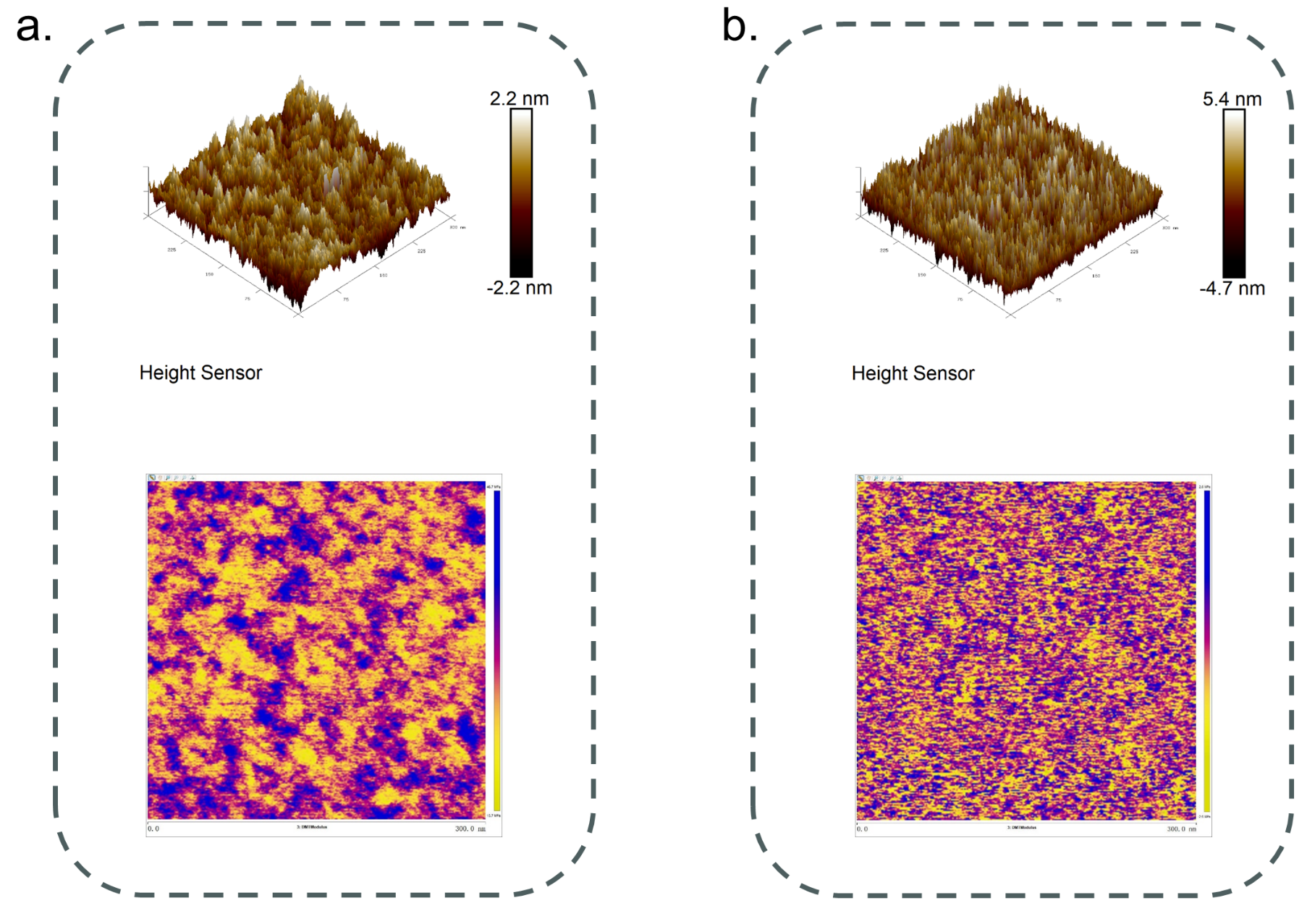


Figure S6. AFM height (i) and modulus (ii) maps of a 300×300 nm^2^ region of (a) SPPU-2 and (b) SPPU-4.


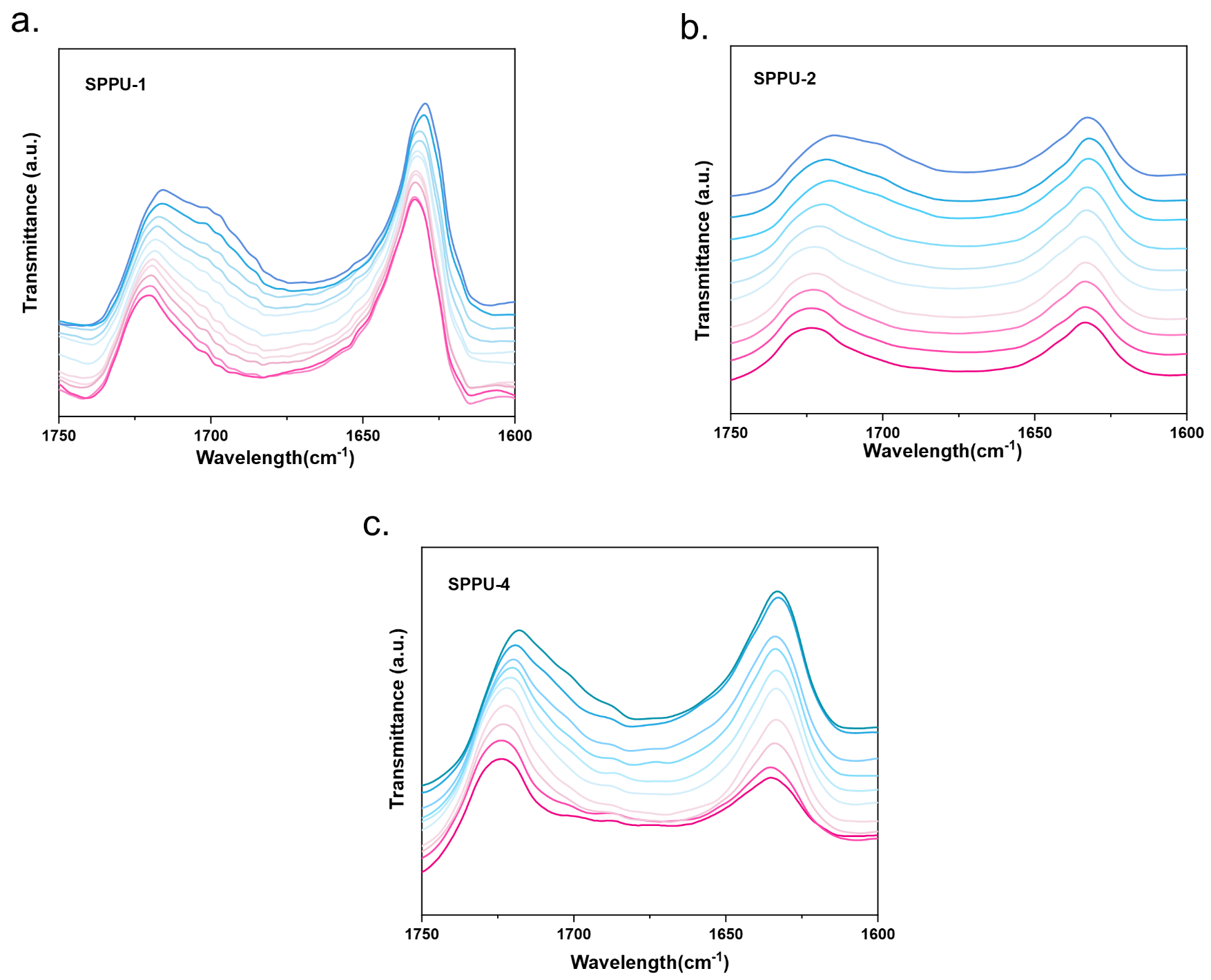


Figure S7. In-situ FTIR of (a) SPPU-1, (b) SPPU-2 and (c) SPPU-4.

It is clear from the Figure S7 that the shoulder peak near 1700 cm-1 becomes progressively more pronounced as the hard segment content increases, attributed to more hydrogen bonds being formed by the hard segment.


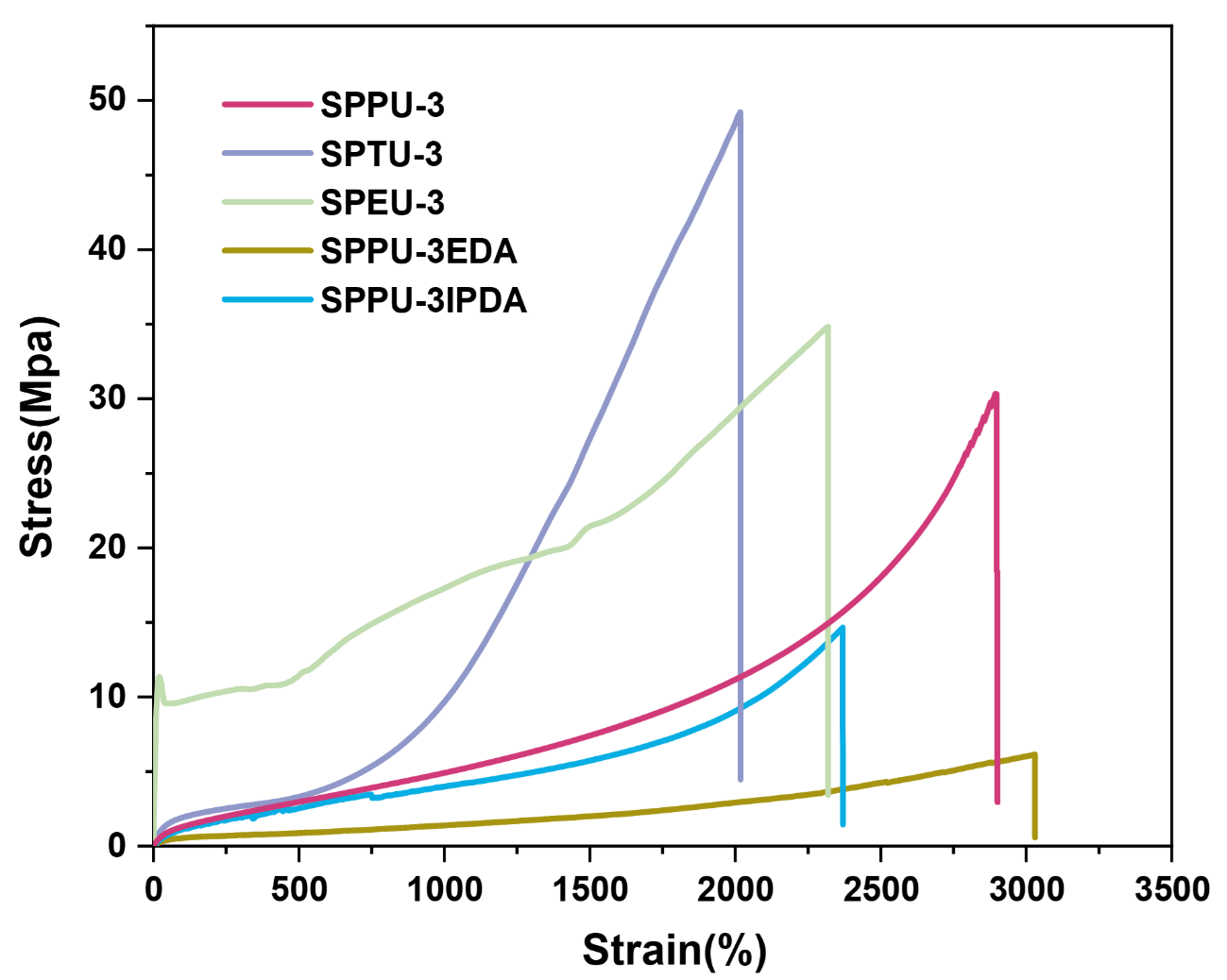


Figure S8. Mechanical properties of SPEU-3, SPTU-3, SPPU-3_IPDA_ and SPPU-3_EDA_.


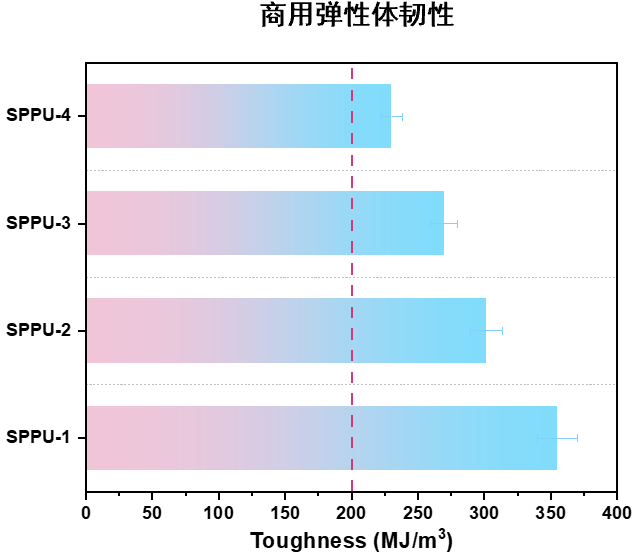


Figure S9. Toughness of SPPUs (the red line in the graph shows the toughness of commercial elastomers)


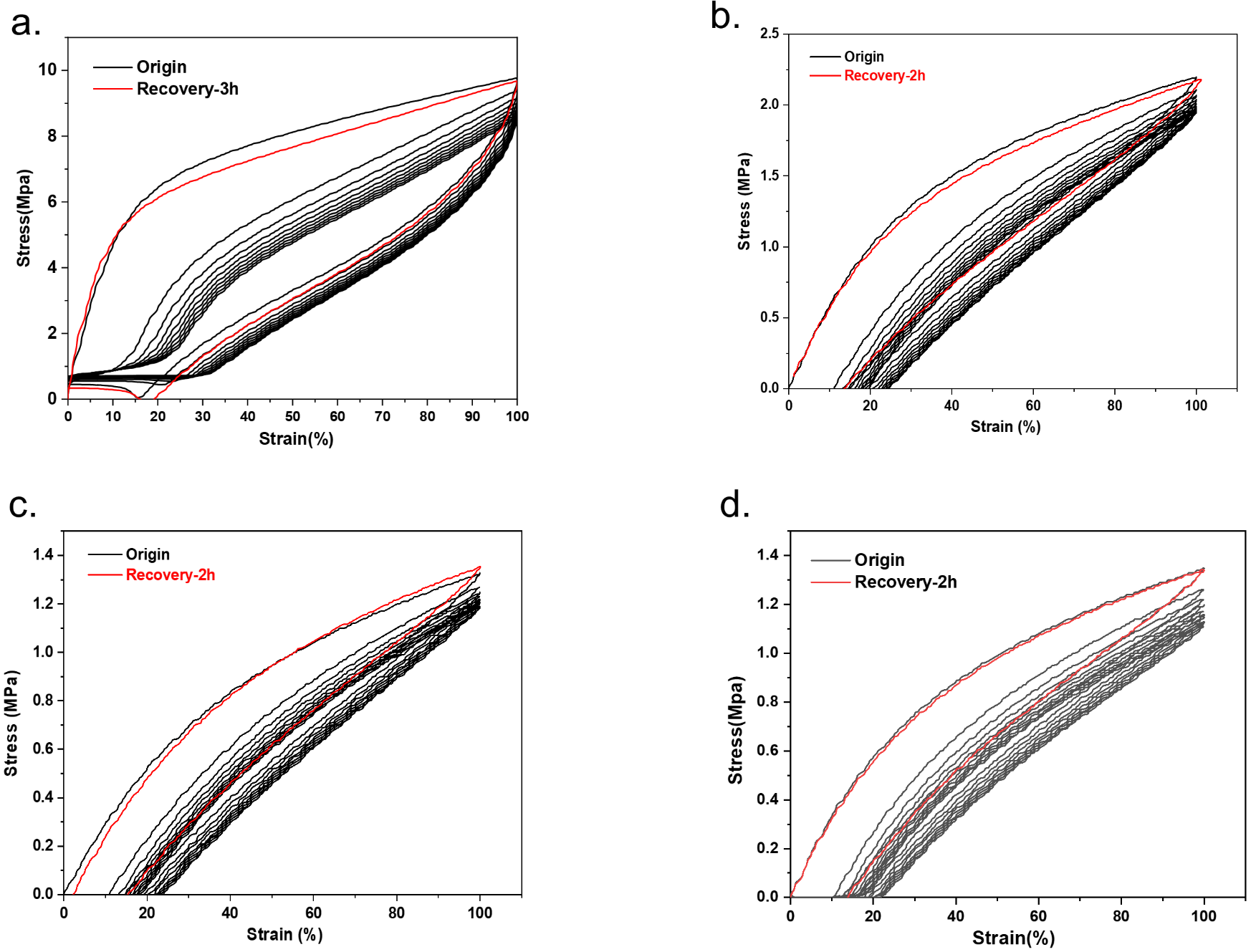


Figure S10. 100% strain cyclic stretching (10 cycles with no intervals)


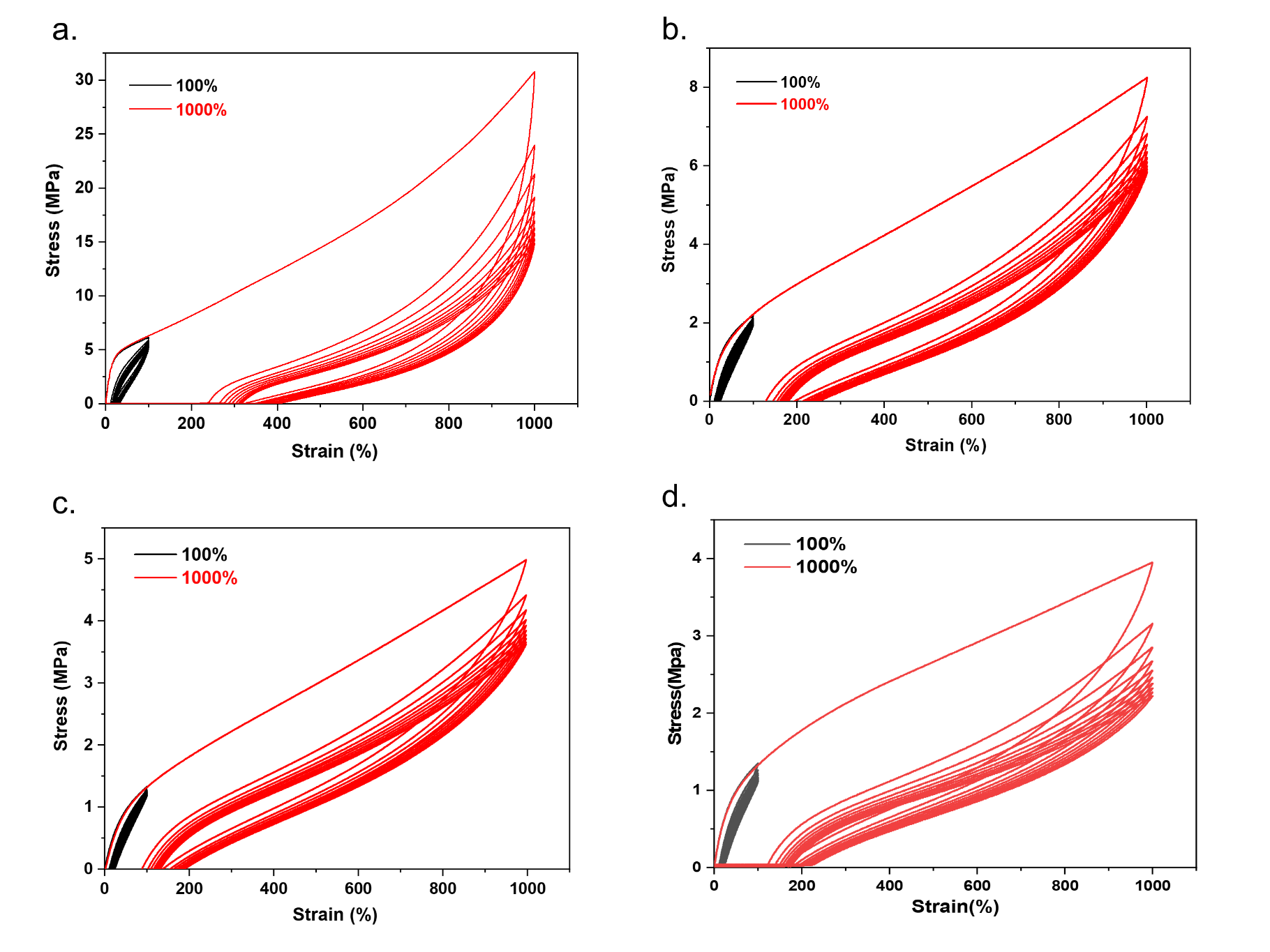


Figure S11. 1000% strain cyclic stretching (10 cycles with no intervals).


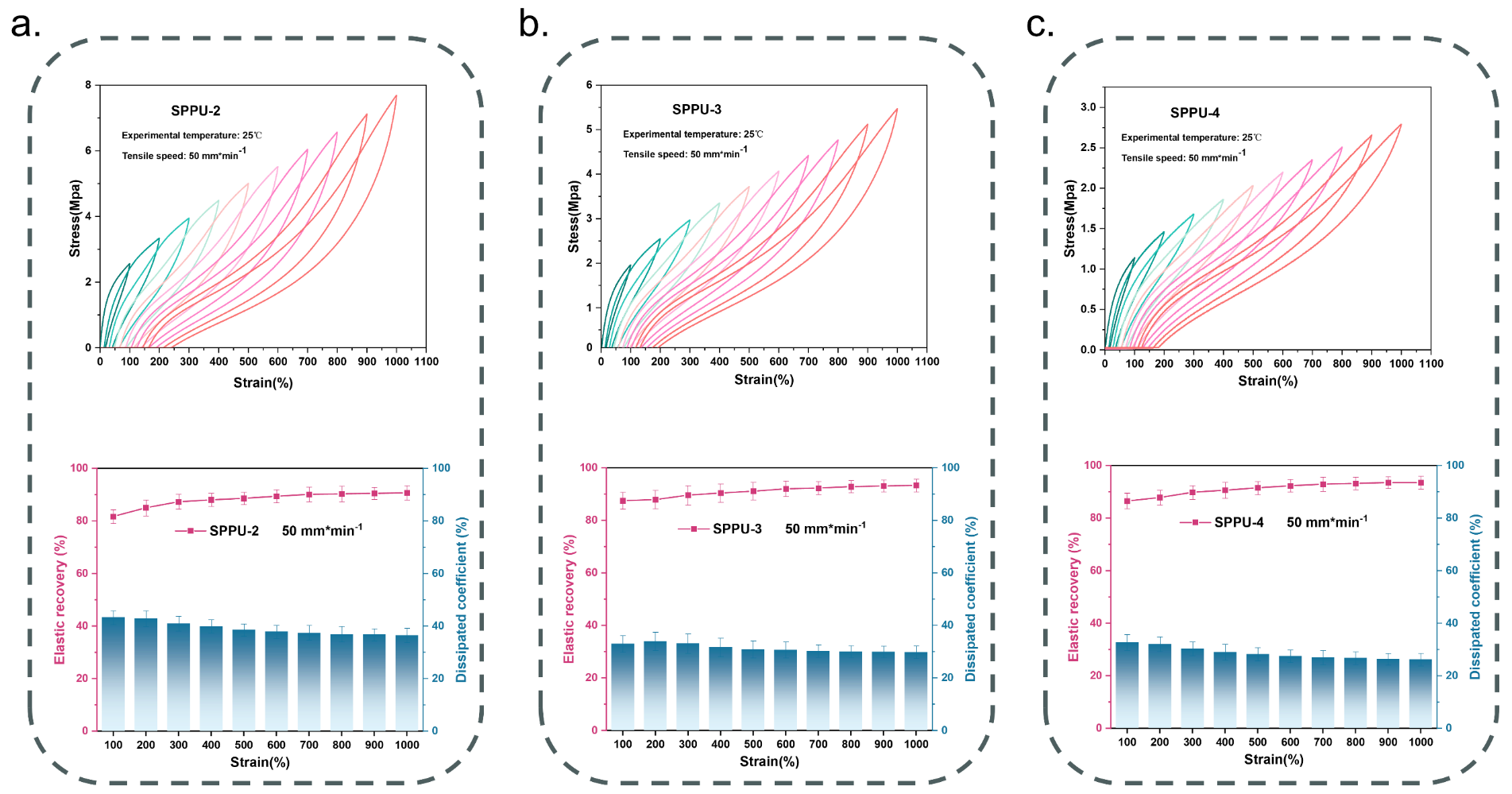


Figure S12. Increasing strain cyclic tensile curves and their Energy Dissipation Index as well as Elasticity Coefficients for (a) SPPU-2, (b)SPPU-3 and (c) SPPU-4.


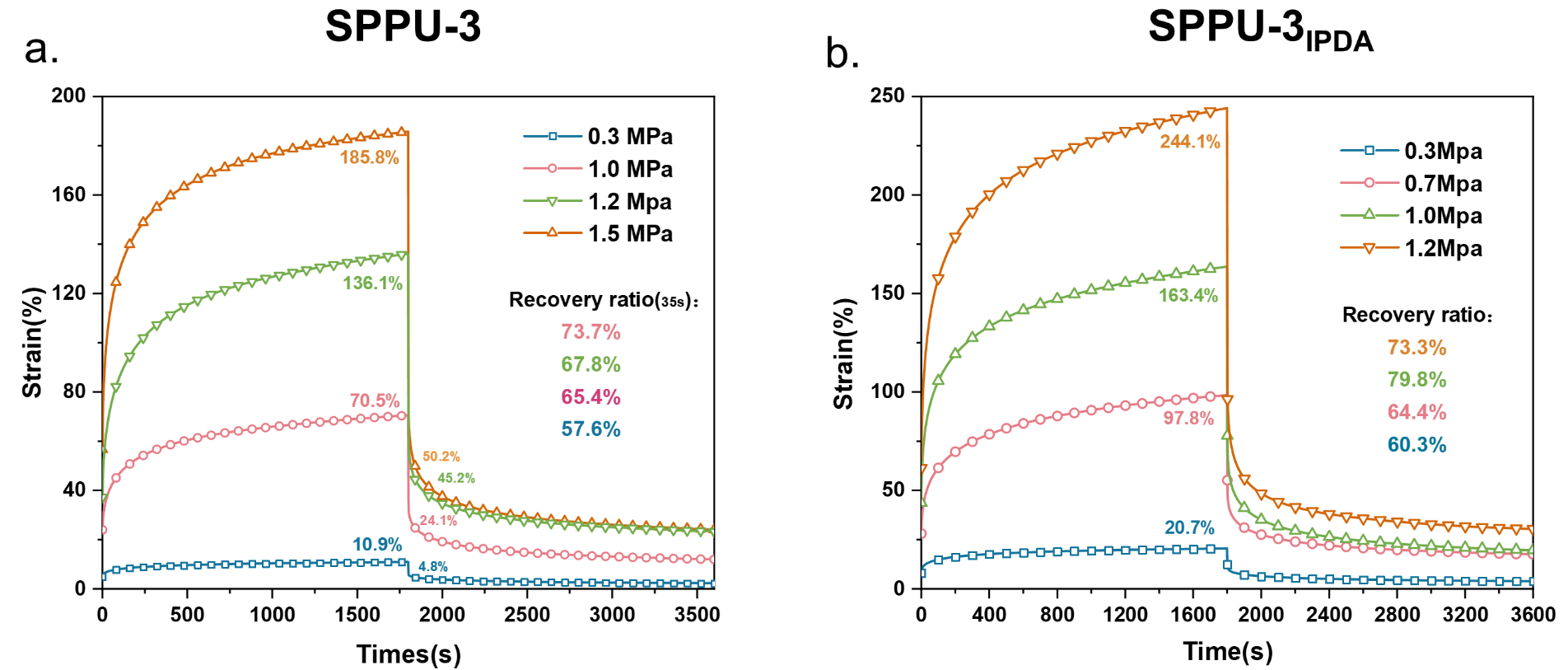


Figure S13. Creep-Recovery curves for varying initial force (a) SPPU-3 and (b) SPPU-3_IPDA_.


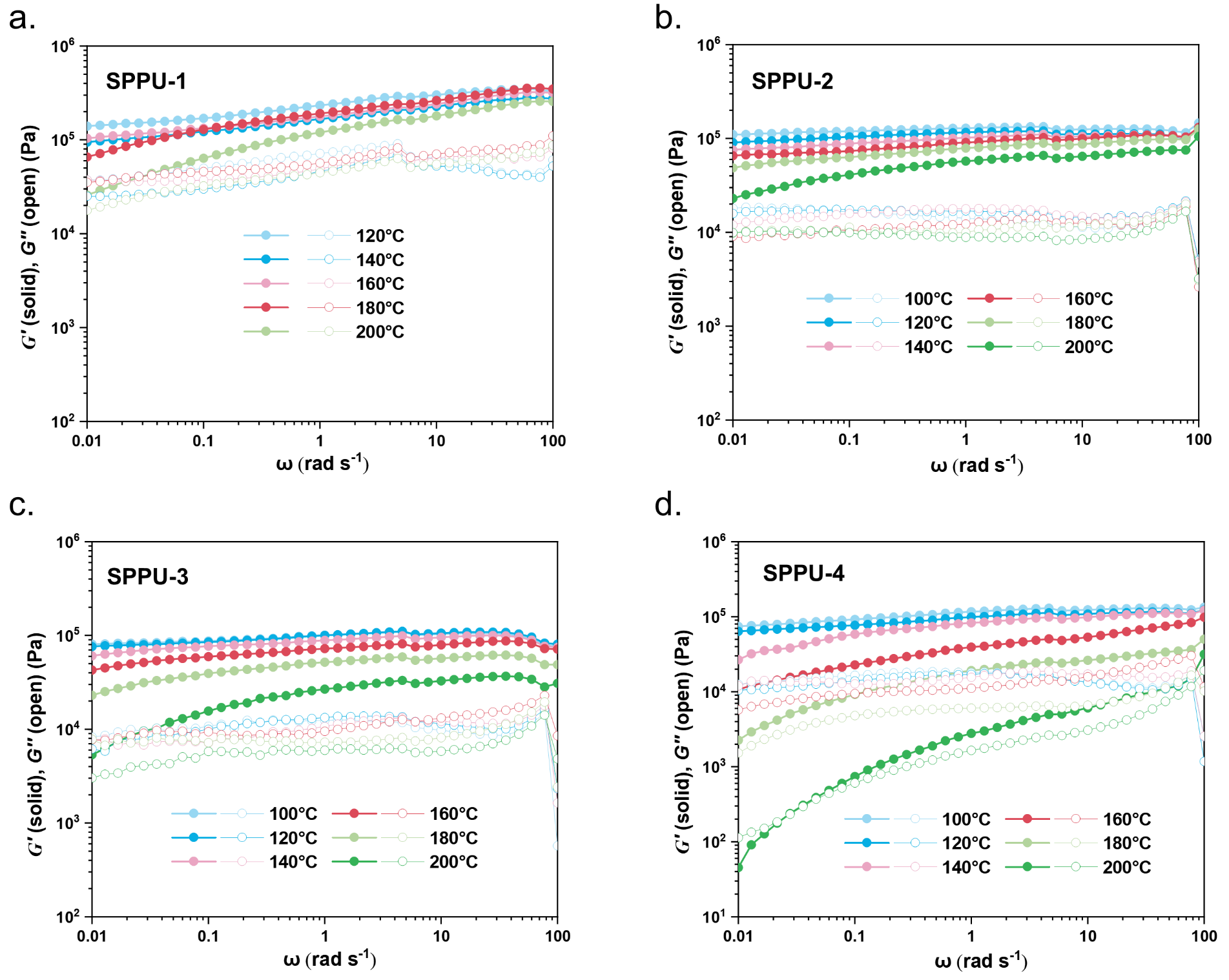


Figure S14. Rheological profile at different temperatures for (a-d) SPPU-1, 2, 3 and 4.


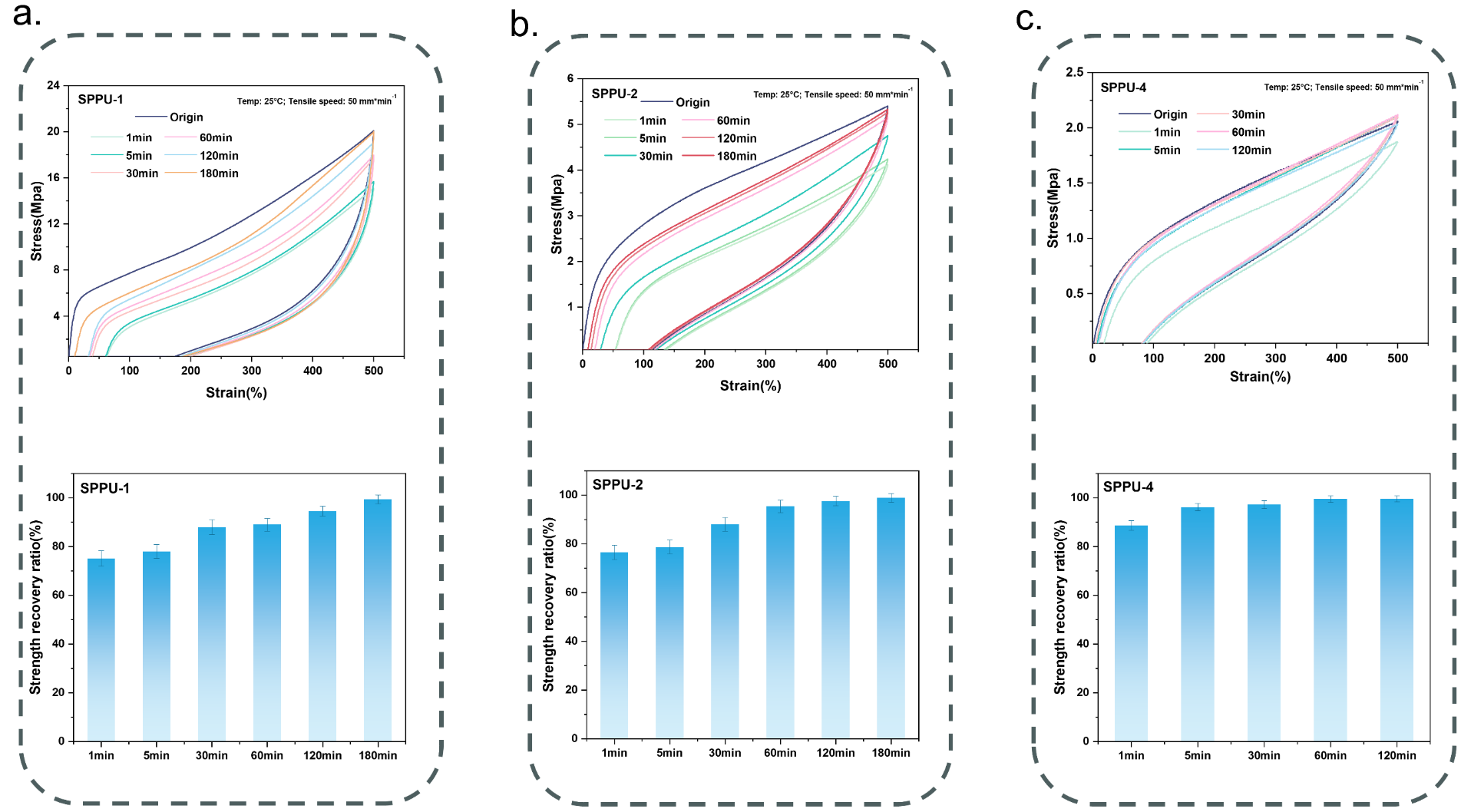


Figure S15. Cyclic stretching with variable recovery time and strength recovery ratio (strain: 500%) for (a) SPPU-1, (b)SPPU-2 and (c) SPPU-4.


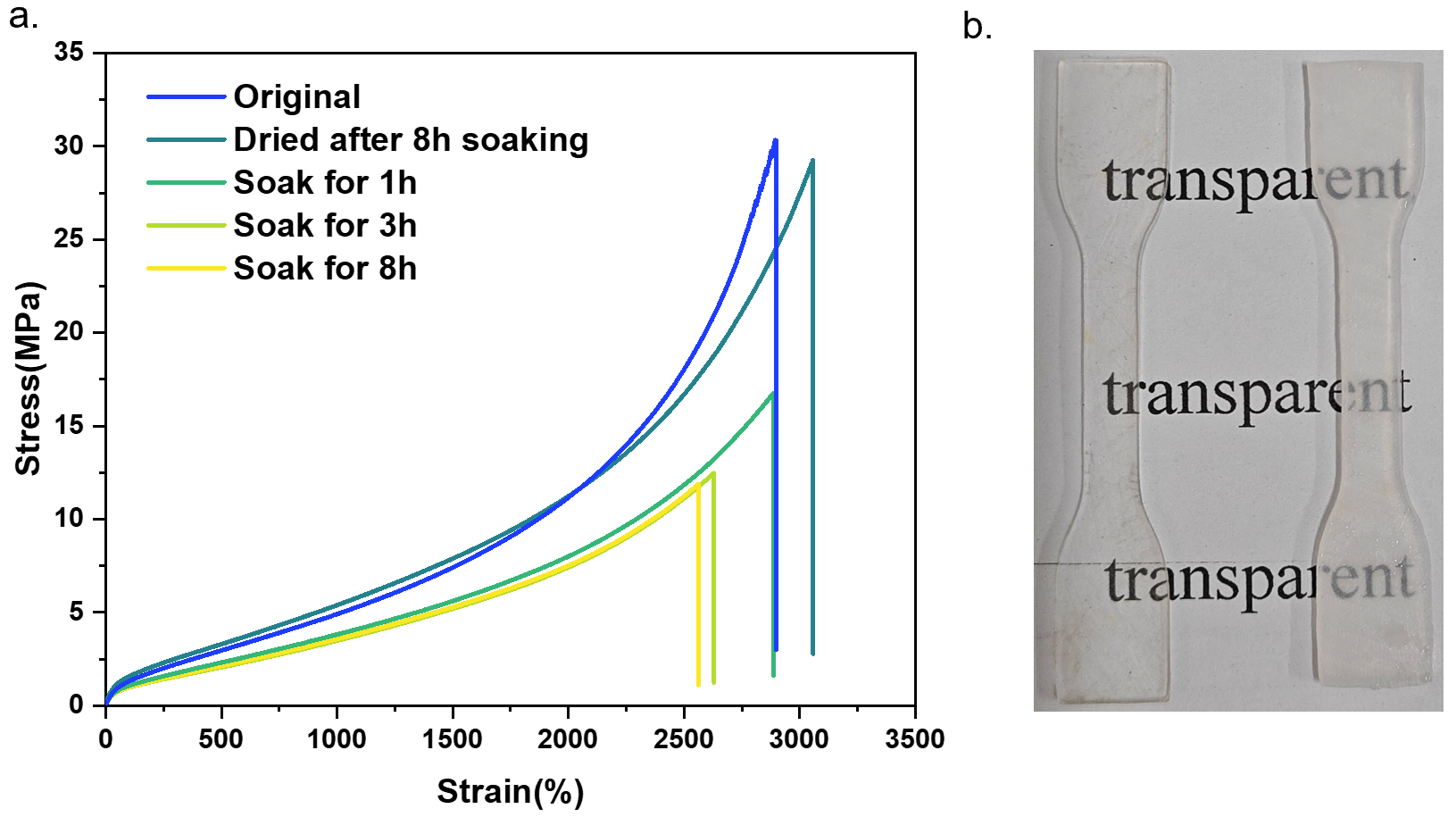


Figure S16. (a) Mechanical curves obtained by varying the immersion time of SPPU-3 in water. (b) Changes in macro-transparency.

As shown in Figure S16, the strength of SPPU-3 decreased to 56% of the initial after immersing it in water for 1h. Continuing to extend the immersion time to 8h, the strength decreased to 39% of the initial and was similar to that of the immersion time of 3h, but the elongation at break did not change much. The macroscopic manifestation is the change in transparency. When the soaked SPPU-3 was dried, its mechanical properties were fully restored, indicating the excellent water resistance of SPPU-3.


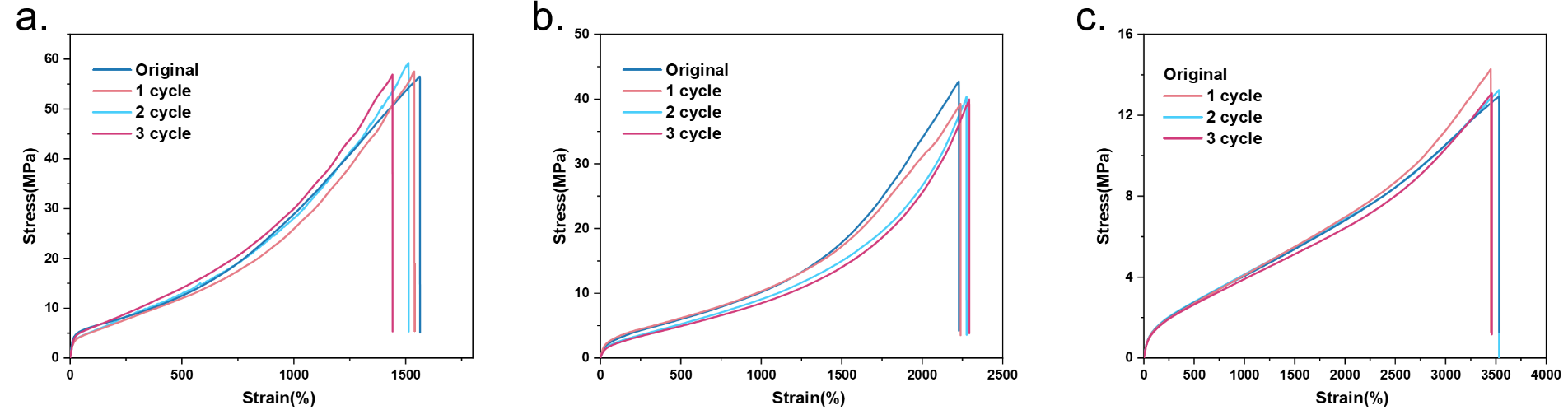


Figure S17. Comparison of mechanical properties before and after alcohol solvent recovery for (a) SPPU-1, (b)SPPU-2 and (c) SPPU-4.


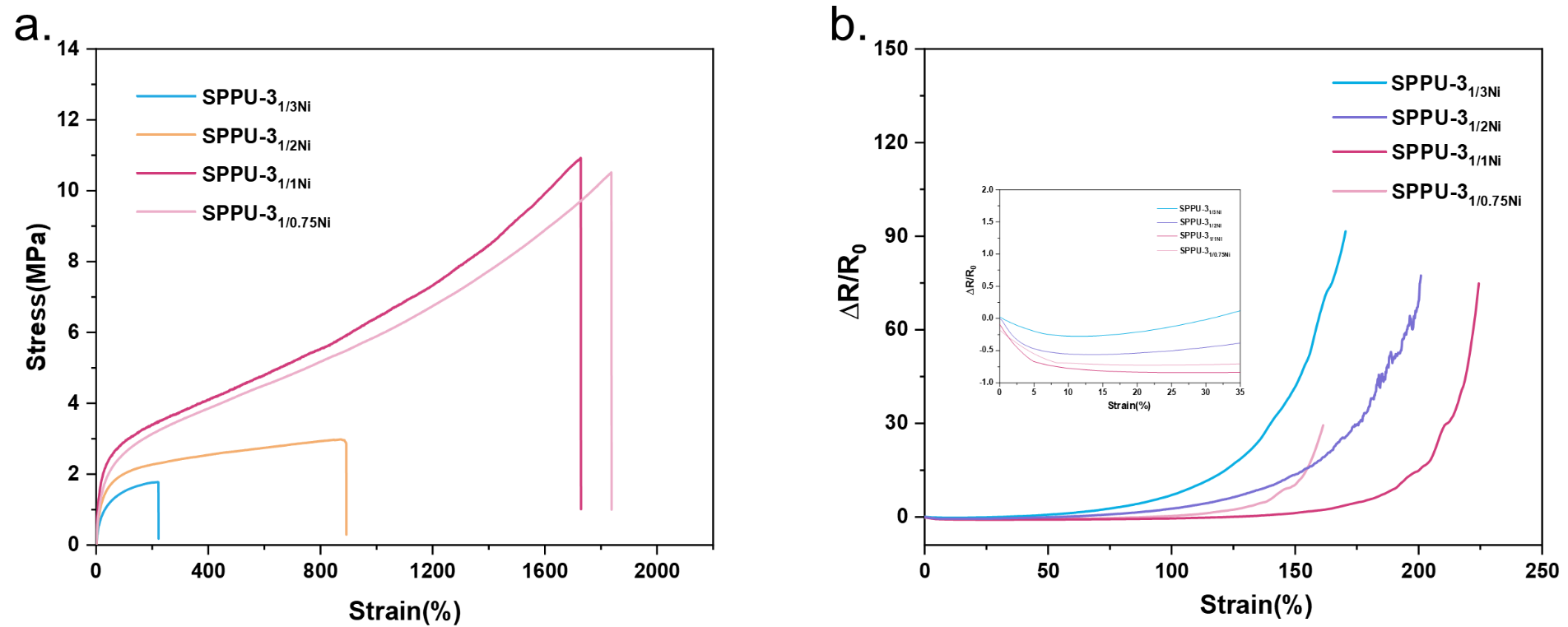


Figure S18. (a) Tensile curves of SPPU-3_1/xNi_ (x=0.75, 1, 2 and 3) and (b) resistance variation with strain increase during stretching.

Accompanying the rapid development of flexible sensors, a large amount of e-waste is incurred, which not only wastes resources (precious fillers, e.g., AgNWs and gold particles) but aggravates white pollution[9]. In consequence, the preparation of efficient and recyclable sensing materials received researchers' attention.

Here, benefiting from the unique room-temperature alcohol solubility of SPPUs, we prepared SPPU-Ni composites with super tensile properties, high flexibility, and excellent electrical conductivity by blending its alcohol solution with inexpensive Ni powder. To balance the recovery and flexibility of flexible sensors, SPPU-3 was selected as the substrate material to be co-mingled with Ni powder to produce SPPU-3_1/xNi_ (X stands for the mass of Ni powder as a multiple of the mass of SPPU-3, X=0.75, 1.0, 2.0 and 3.0). Compared with the original SPPU-3, the mechanical properties suffered a certain degree of degradation with the addition of Ni powder, whereas it remained decent strength and toughness. As shown in Figure S18, we measured the resistance changes of SPPU-3 with different Ni powder contents during the tensile process, respectively. In terms of mechanical properties, as the Ni powder content was increased, the mechanical properties remained stable at the initial stage, but decreased dramatically when the mass of the filler reached two times the mass of the substrate material (strength of 3 MPa, elongation at break of 880%). Continuing to increase the Ni content to 3 times the mass of the substrate material, the strength decreased to 1.8 MPa and the elongation was 221%, but still possessing excellent flexibility. The reasons for the decrease in mechanical properties can be acquired from SEM and EDS (as shown in Figure S19-22). When the Ni powder content is low (X<2), the substrate materials are closely connected to each other, which ensures satisfactory tensile properties (elongation at break >1600%, and strength >10MPa). After the Ni powder content is elevated to 2, a substantial amount of Ni powder not only segregates the connection between the substrate materials, it also wraps the air to form a cavity inside the material, causing numerous defects inside the material, rendering a significant decrease in the mechanical properties.


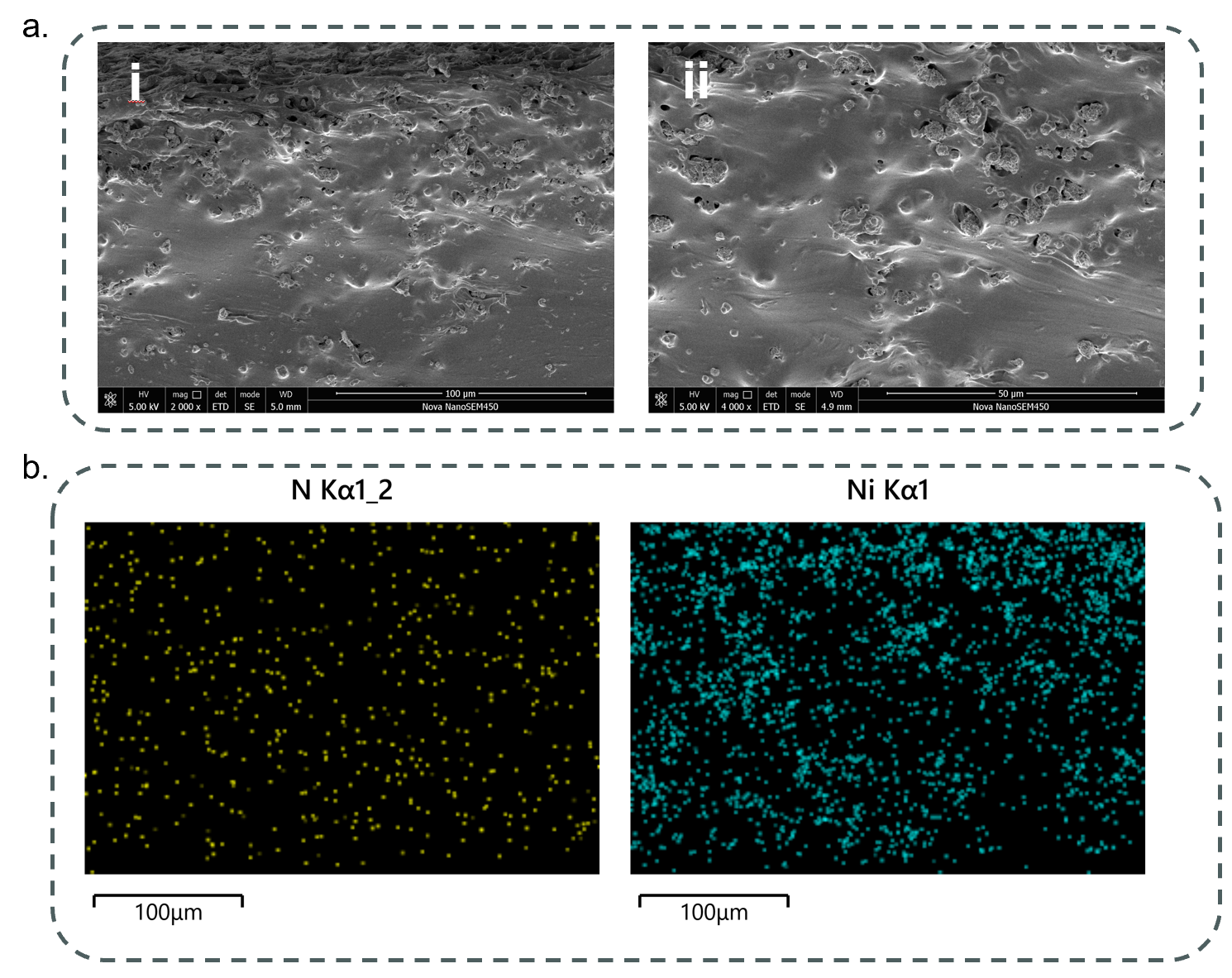


Figure S19. (a) SEM of SPPU-3_1/0.75Ni_, (b) EDS of SPPU-3_1/0.75Ni_. (Bottom surface at high content of Ni element)


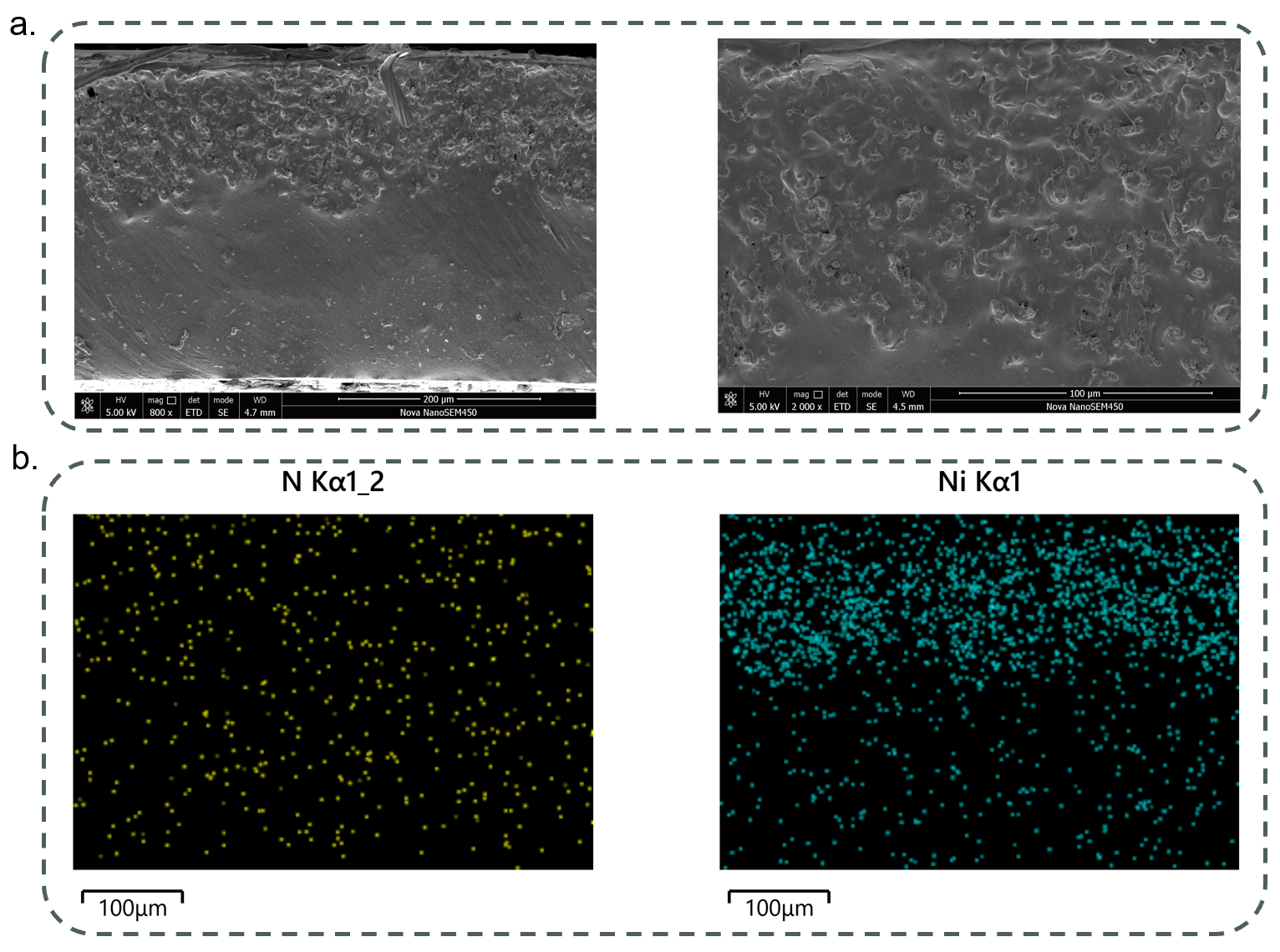


Figure S20. (a) SEM of SPPU-3_1/1Ni_, (b) EDS of SPPU-3_1/1Ni_ (using i in (a) as a template).


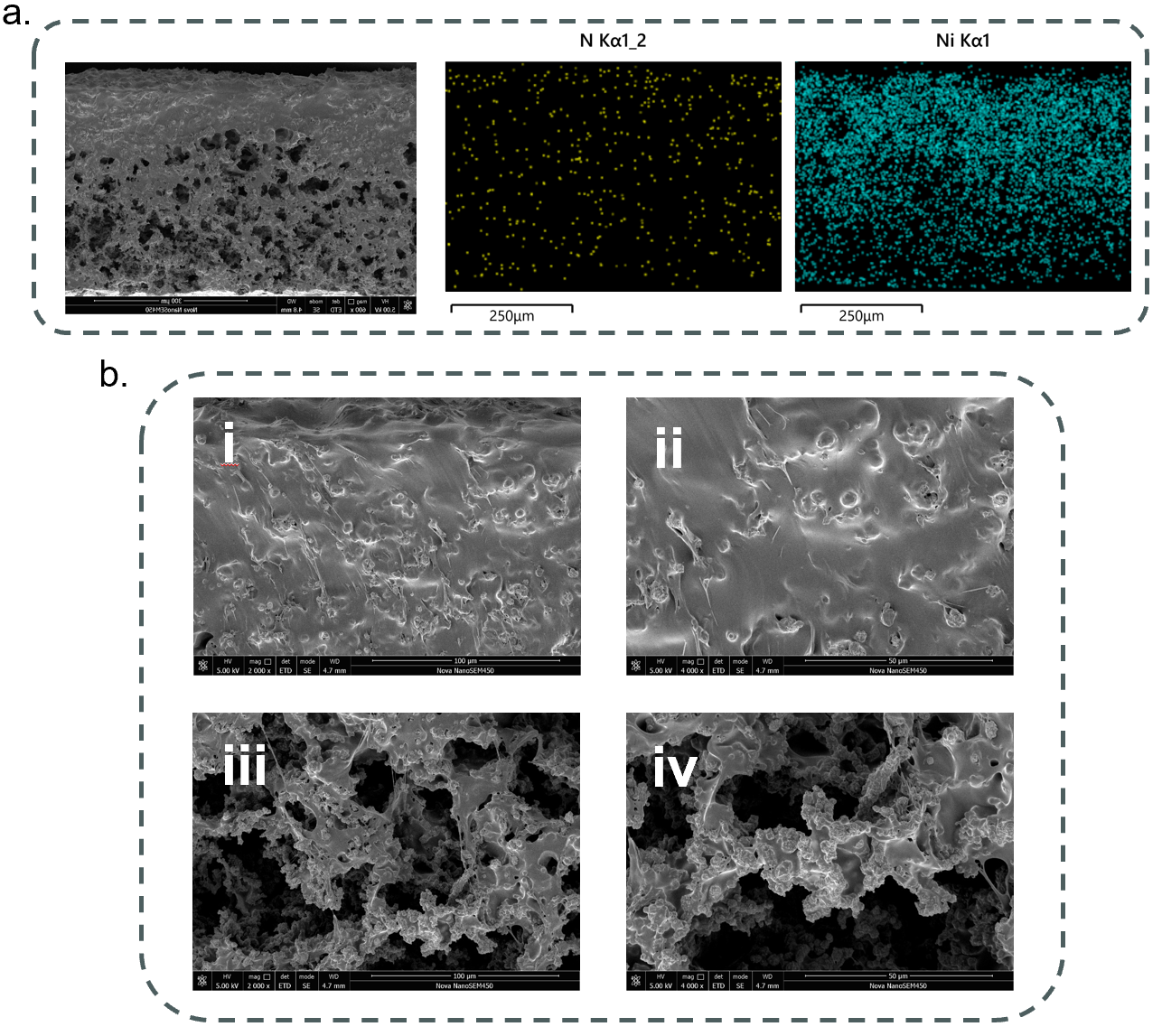


Figure S21. (a) SEM of SPPU-3_1/2Ni_ and its EDS images, (b) SEM of SPPU-3_1/2Ni_.


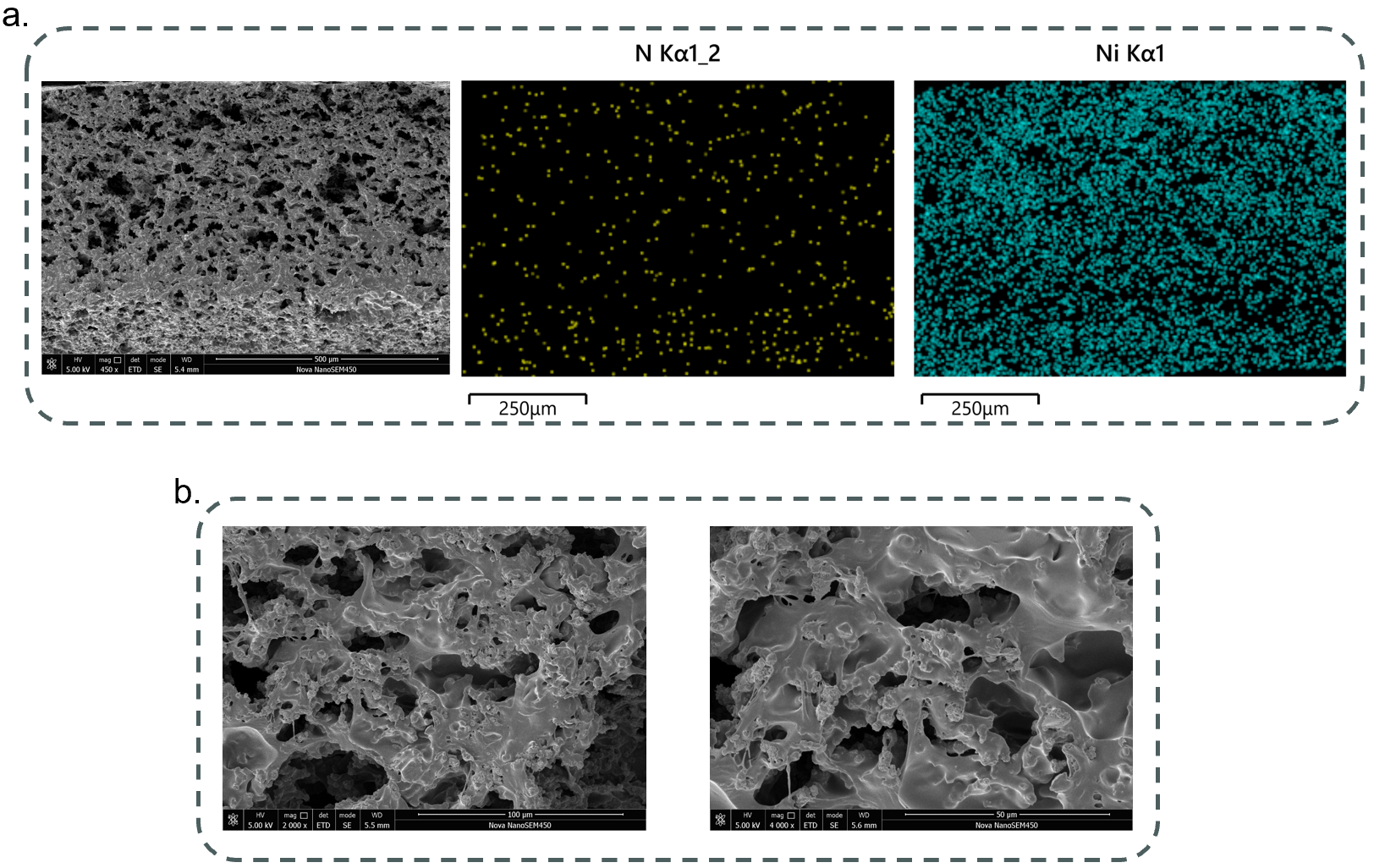


Figure S22. (a) SEM of SPPU-3_1/3Ni_ and its EDS images, (b) SEM image SPPU-3_1/3Ni_.


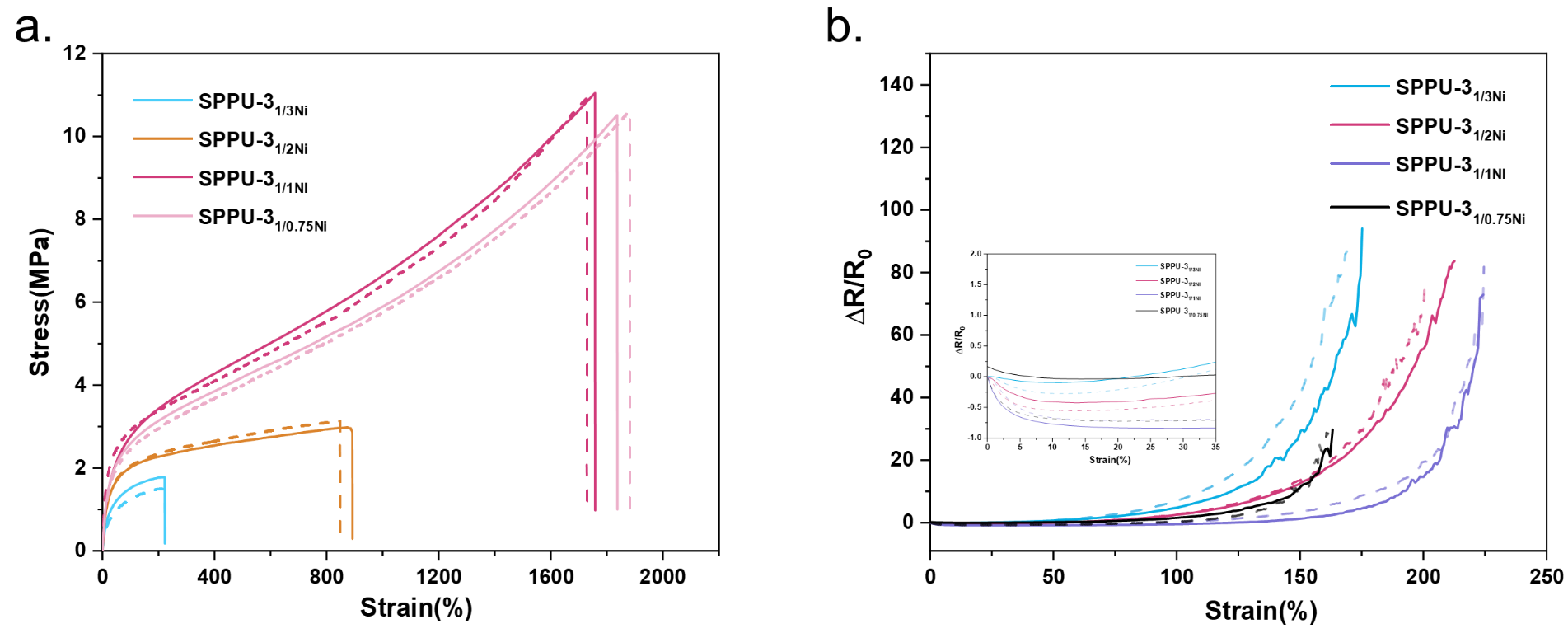


Figure S23. Comparison of (a) tensile curves and (b) strain-resistance variation curves (the dotted line represents the recovered) before and after recovery of SPPU-3_1/xNi_ (x=0.75, 1, 2 and 3).

At the resistance change level, we noticed a consistent feature for all samples, where the resistance initially decreases during the initial stretching phase (around 15%), followed by a slow increase and finally a sharp increase. And the resistance change in the initial stage is relevant to Ni powder content, as shown in Figure S23a and S23b, and this phenomenon is gradually apparent with the decrease of Ni powder content. This may be attributed to the existence of two competing effects during the initial stretching process, the decrease in cross-sectional area from stretching by an external force and the movement along the stretching direction. In the initial stage, the Ni powder in SPPU-3_1/xNi_ is mainly subjected to the effect of cross-sectional area reduction, which allows more contact between the conductive fillers and improves the conductivity. And the fewer the initial conductive pathways are more sensitive to this effect (sensitivity: SPPU-3_1/0.75Ni_> SPPU-3_1/3Ni_). When stretching continues, Ni powder moves in the direction of stretching under the force, and the conductive pathway diminishes until the final pathway disappears[10].


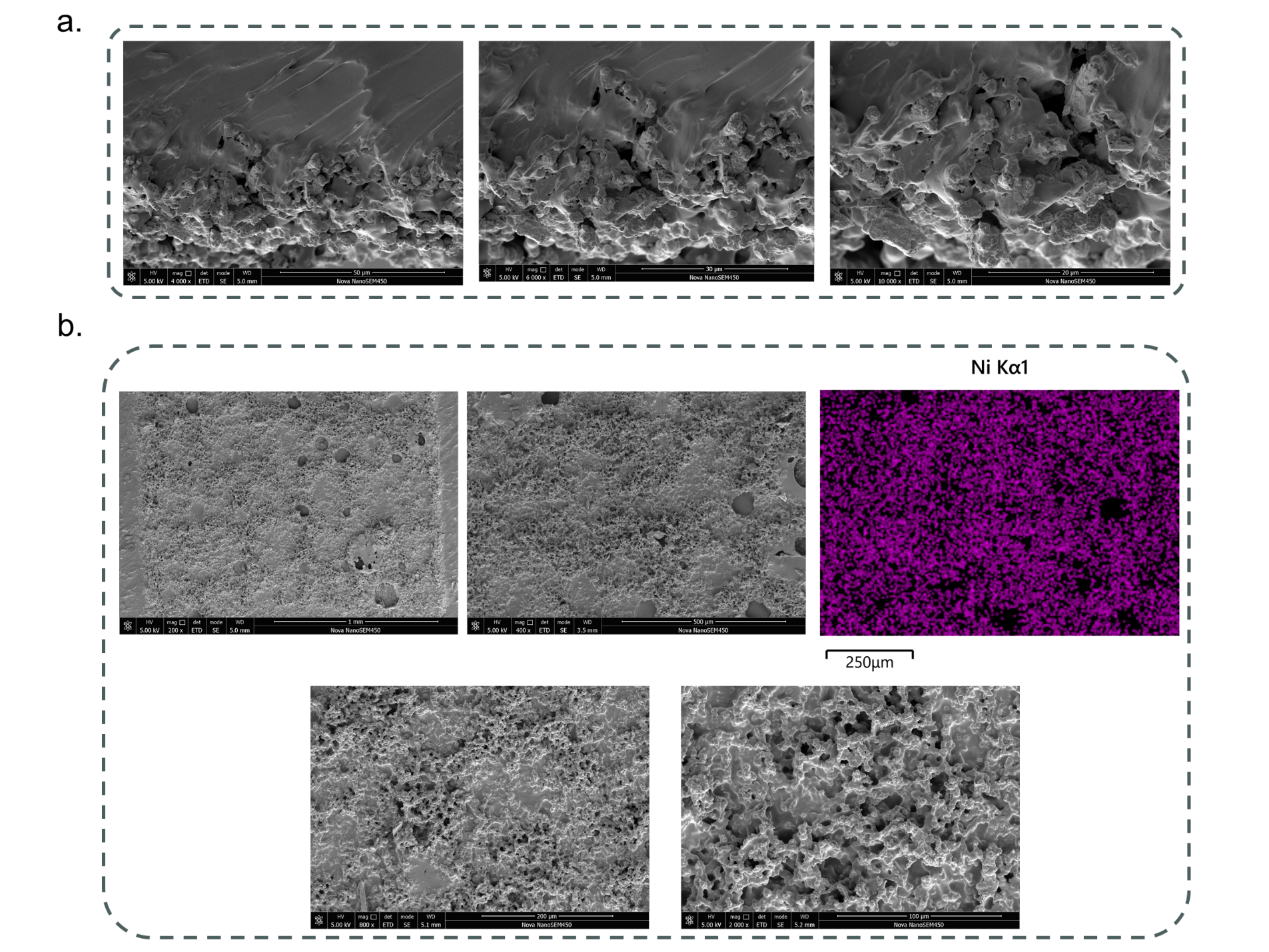


Figure S24. (a) SEM image of the cross-section as well as (b) SEM and EDS of the surface of capacitor lines based on SPPU-3_1/3Ni_ paste printed on SPPU-3.

Firstly, we observe the cross-section of the capacitor device with SPPU3 as the substrate material. We found that the circuits printed on SPPU3 are tightly connected with the substrate material because the molecular chains of SPPU3 will start to move under the action of ethanol in the slurry and entangle with the molecular chains in the slurry, and the final circuits formed are integrated with the substrate material. Subsequently, we can also find that the Ni powder is uniformly distributed and formed pathways from the SEM as well as EDS pictures of the surface.


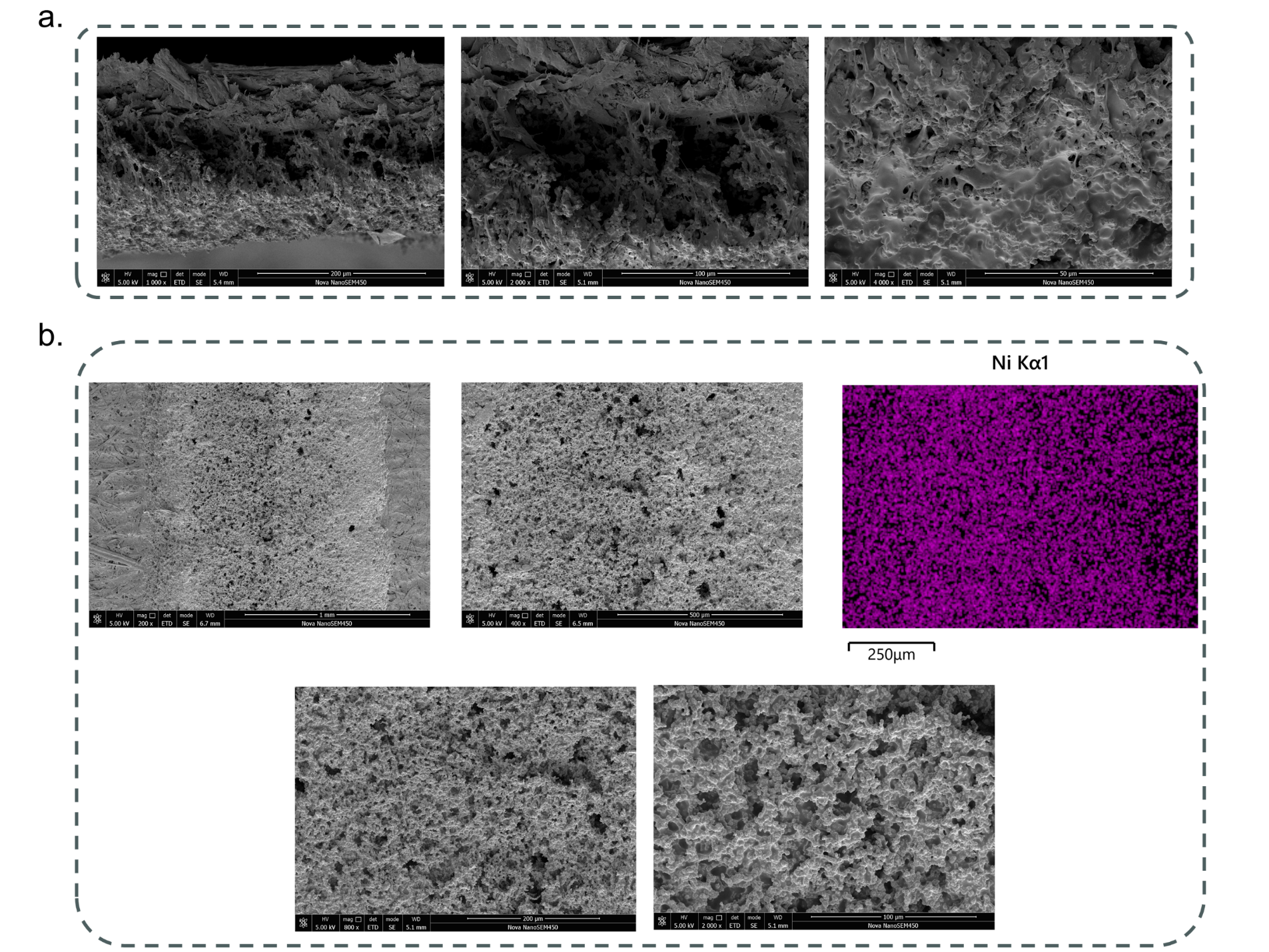


Figure S25. (a) SEM image of the cross-section as well as (b) SEM and EDS of the surface of capacitor lines based on SPPU-3_1/3Ni_ paste printed on paper.

First, we investigated the cross-section of a capacitor device with paper as the substrate material. It was found that the circuits printed on the paper have a good connection with the substrate material due to the rough structure of the paper surface which allows the paste to be in full contact with it as well the possible presence of hydrogen bonding to promote the bonding of the two. Subsequently, we can also notice that in the SEM and EDS photographs of the surface, the Ni powder is uniformly distributed and has a higher pathway content than the SPPU3-based capacitor devices, which is characterized by a stronger capacitive signal.


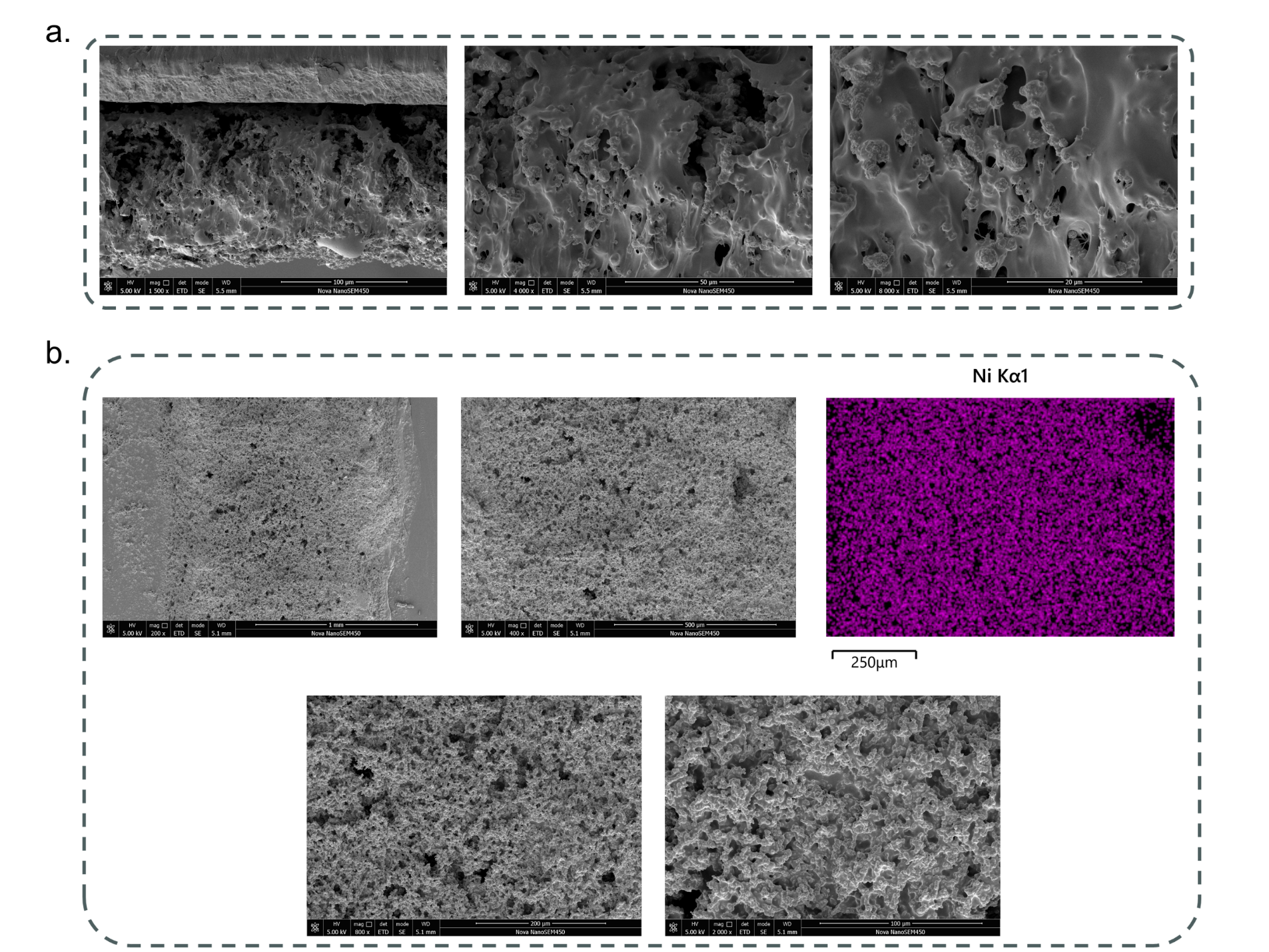


Figure S26. (a) SEM image of the cross-section as well as (b) SEM and EDS of the surface of capacitor lines based on SPPU-3_1/3Ni_ paste printed on PET film.

Similar to the previous one, the SEM and EDS images of the capacitor printed on PET film are presented in the Figures. In the cross section it can be noticed that the capacitor lines show a decent connection to the PET. On the surface it also exhibits a uniform distribution of Ni powder and a large number of conductive pathways. The excellent transmittance, flexibility and solvent resistance of PET make it the best choice.


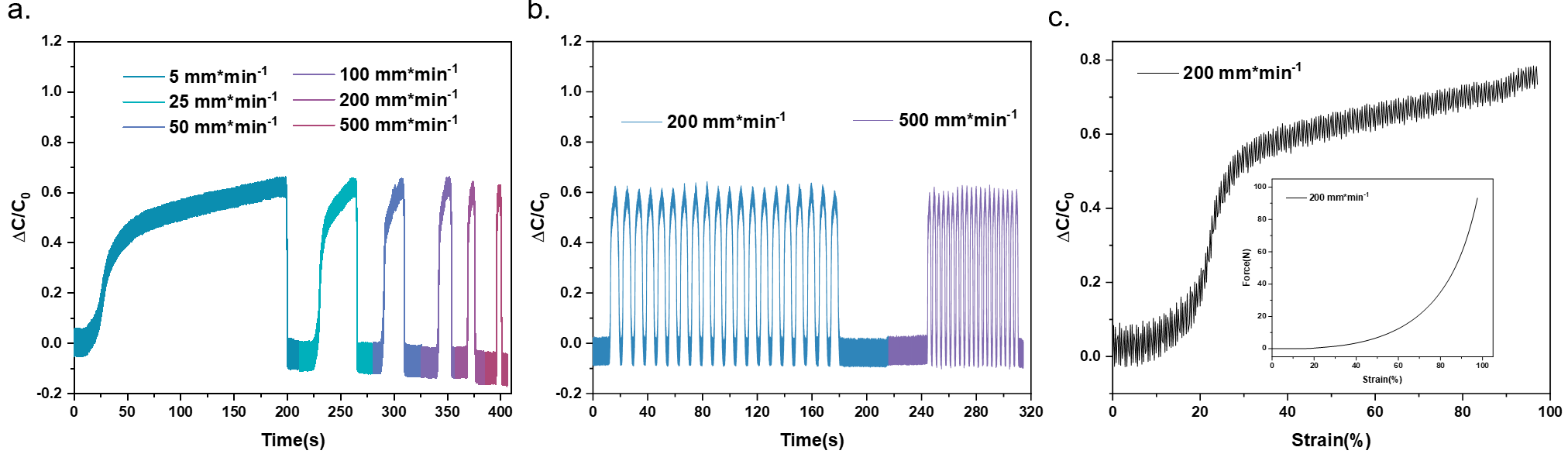


Figure S27. (a) Signal change of the capacitance sensor when varying the compression rate (75% compression); (b) cyclic compression of the capacitance sensor (75% compression); and (c) signal change of the capacitance sensor during compression (95% compression), inset showing the compression strength.

First, we keep the degree of the compression at 75% stationary and continuously change the compression rate obtaining Figure S27a. Even when the compression rate is increased to 500 mm min^-1^, the capacitive sensor still achieves identical signals to the lower rate, demonstrating its excellent sensing stability. Figure S27b illustrates the response of the capacitive sensor at different compression rates (compression level: 75%), demonstrating that it possesses a stable cycling performance. Even under high-speed compression cycling, the capacitive sensor maintains a stable and consistent response signal. Finally, we increase the compression to 95% to acquire Figure S27c. Since a change in capacitance occurs when an external object comes into contact with a capacitive sensor and the degree of signal change is positively correlated with the effective contact area. For example, we can see from the signal-strain curve that there is a rapid rise in signal during initial compression, followed by a slower change attributed to the rapid increase in the effective area at the onset of the compression.


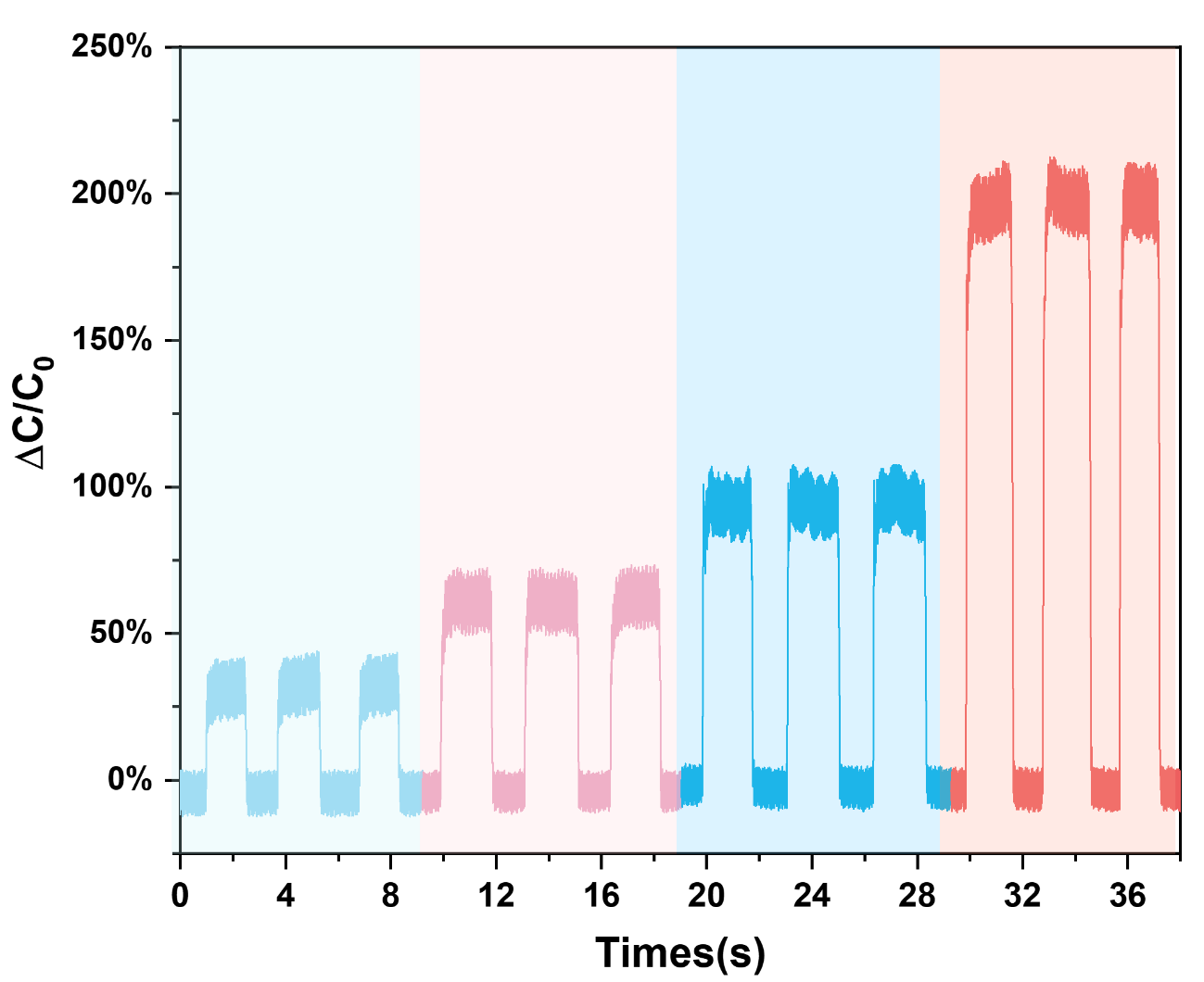


Figure S28. Change in capacitance of PCBS printed on PET film as a function of the number of fingers (increasing press time).


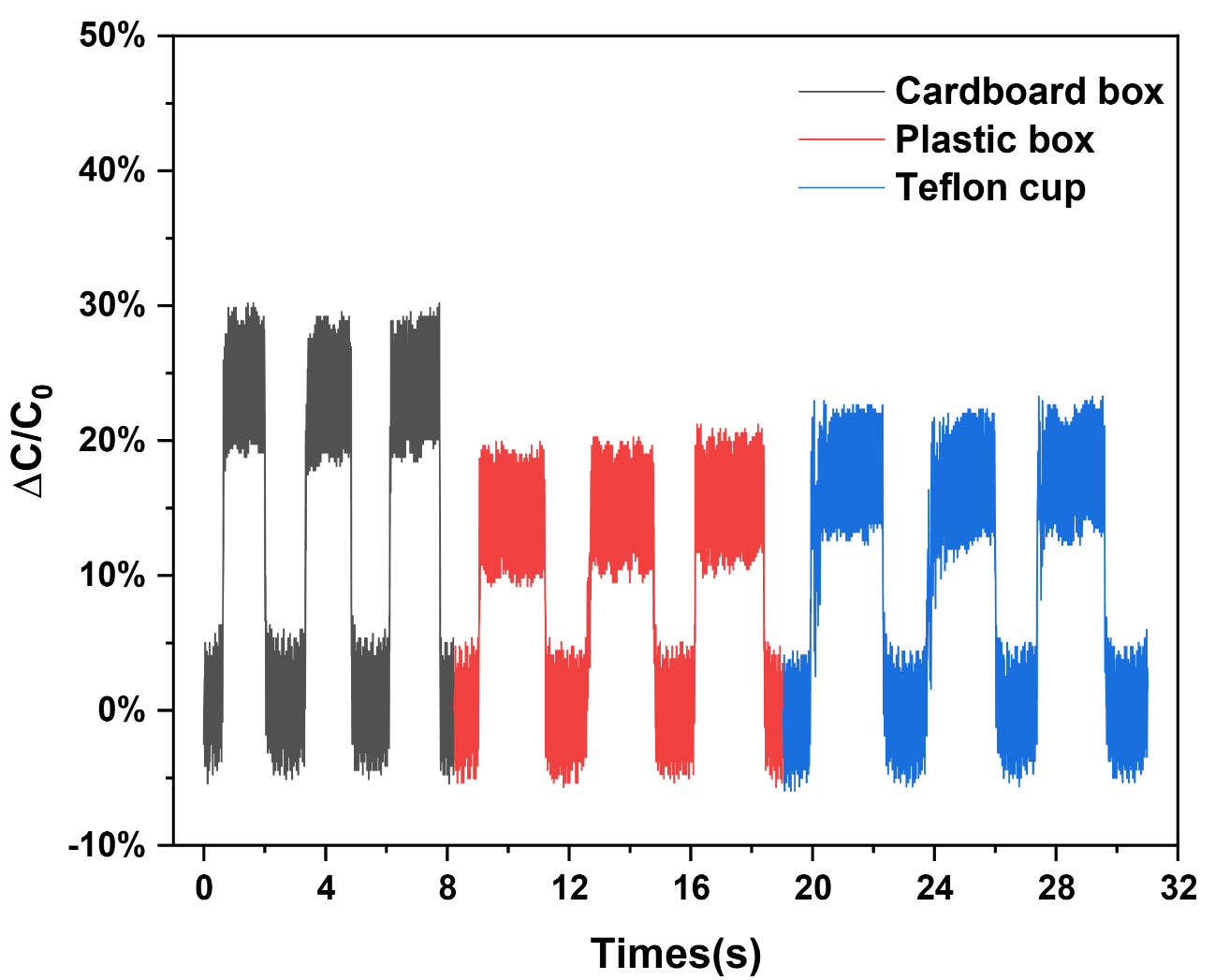


Figure S29. Recognizing different objects in contact using PCBS.


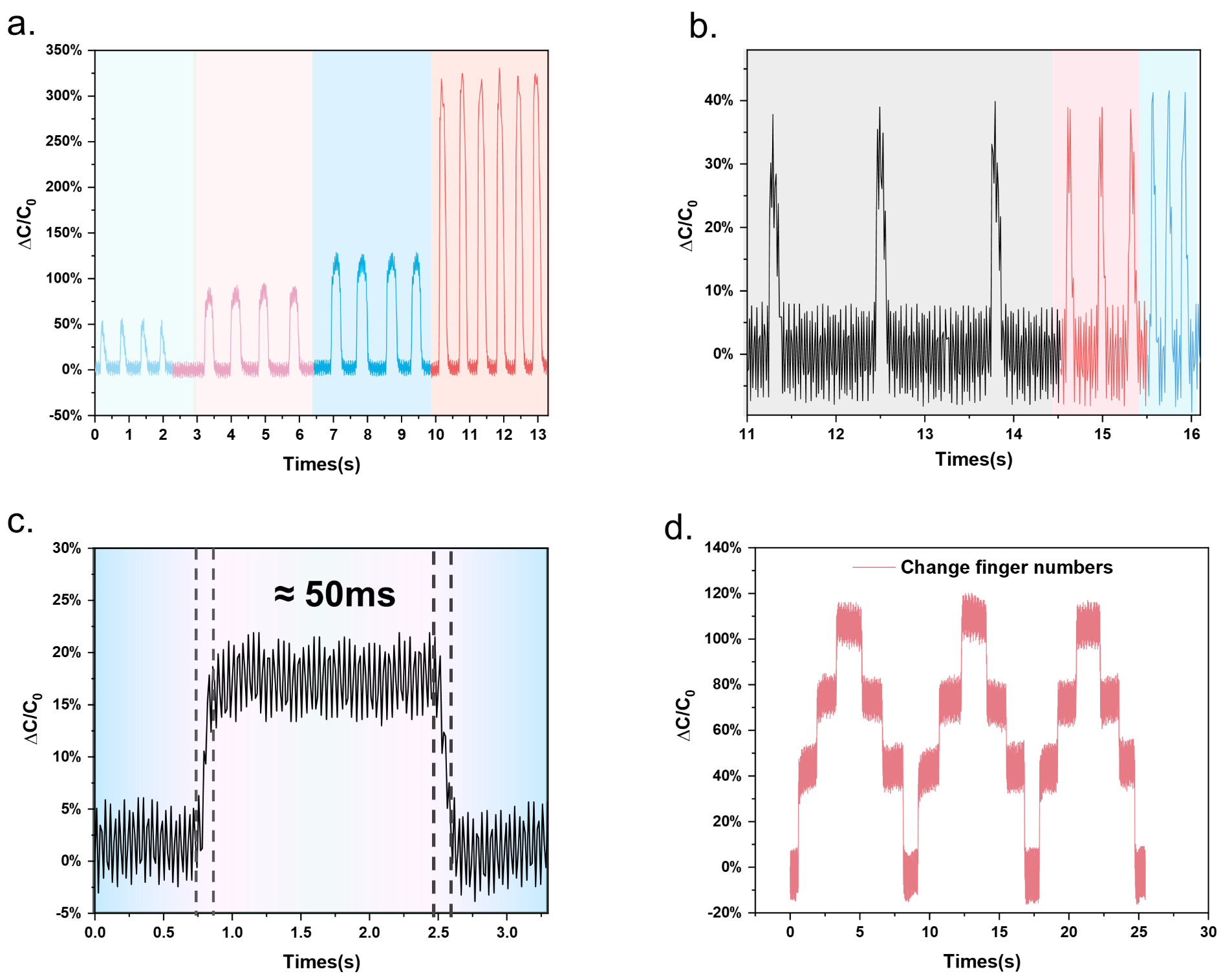


Figure S30. Sensing performance of circuits printed on paper (a) Changing the number of pressing fingers. (b) Changing the frequency of presses (1Hz, 2.5Hz and 5Hz). (c) Response time (≈50ms). (d) Increment/decrement finger count cycles.


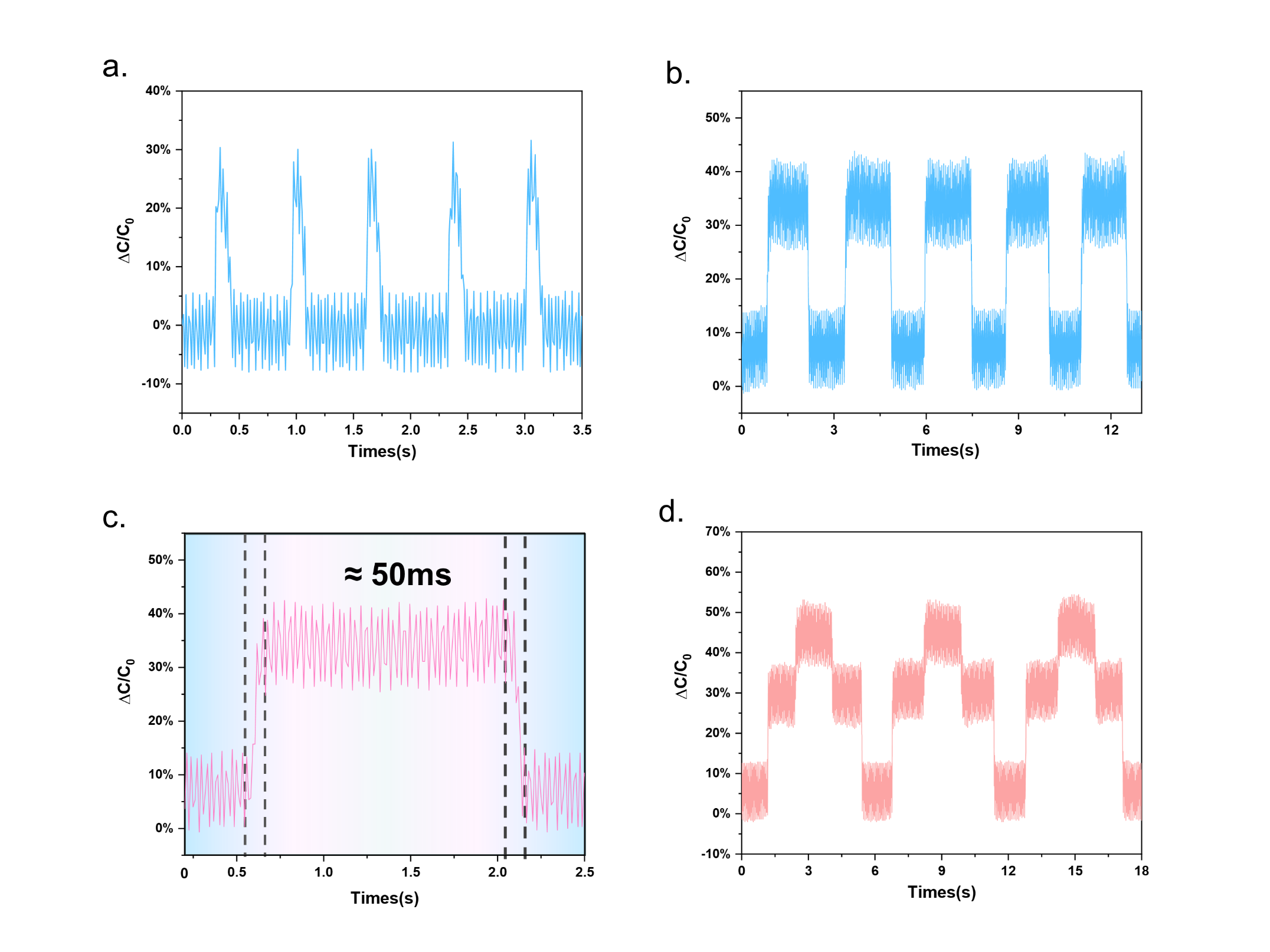


Figure S31. Sensing performance of circuits printed on SPPU3 (a) short press. (b) long press. (c) Response time (≈50ms). (d) Increment/decrement finger count cycles.


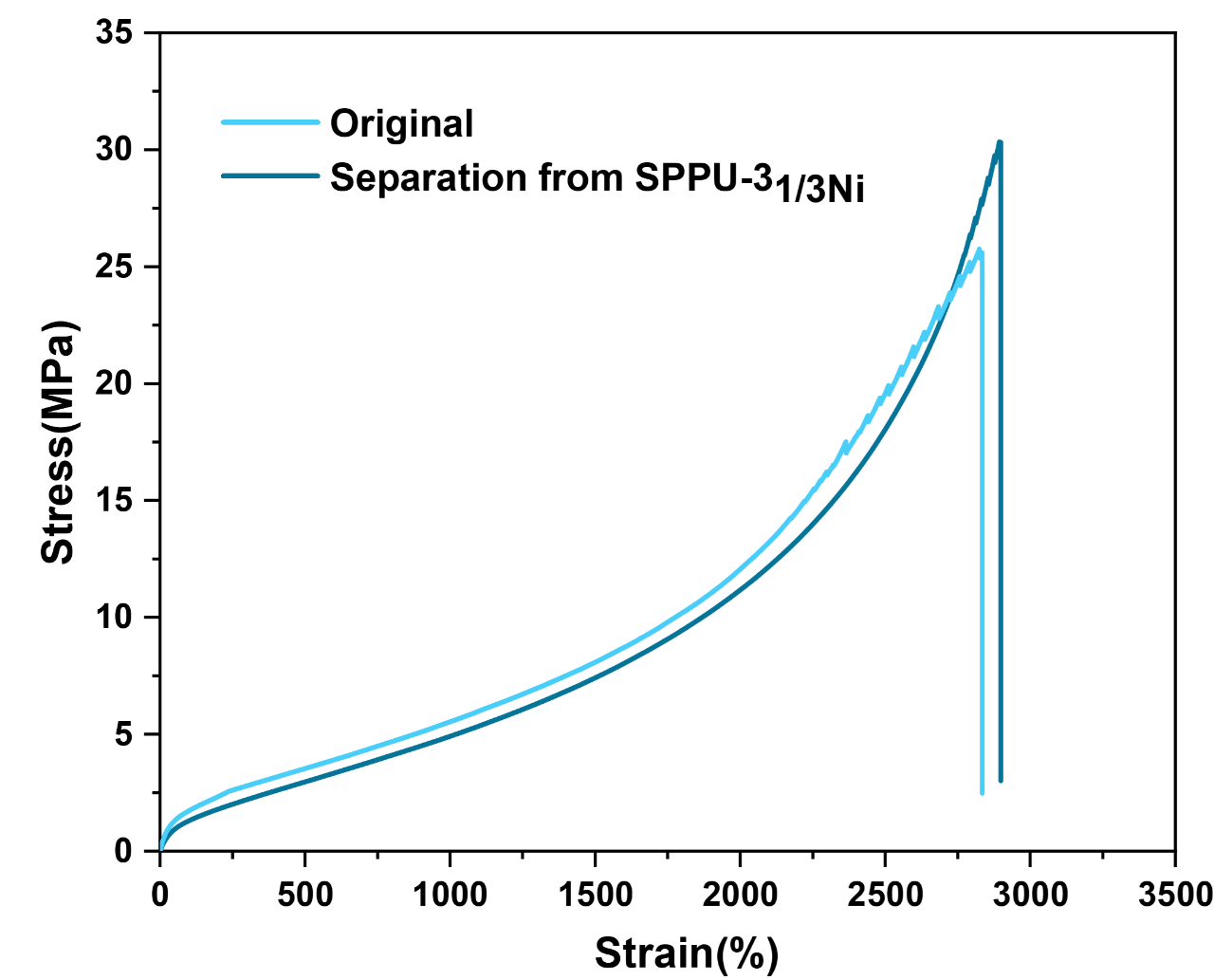


Figure S32. Tensile curve of SPPU-3 recovered from SPPU-3_1/3Ni_.


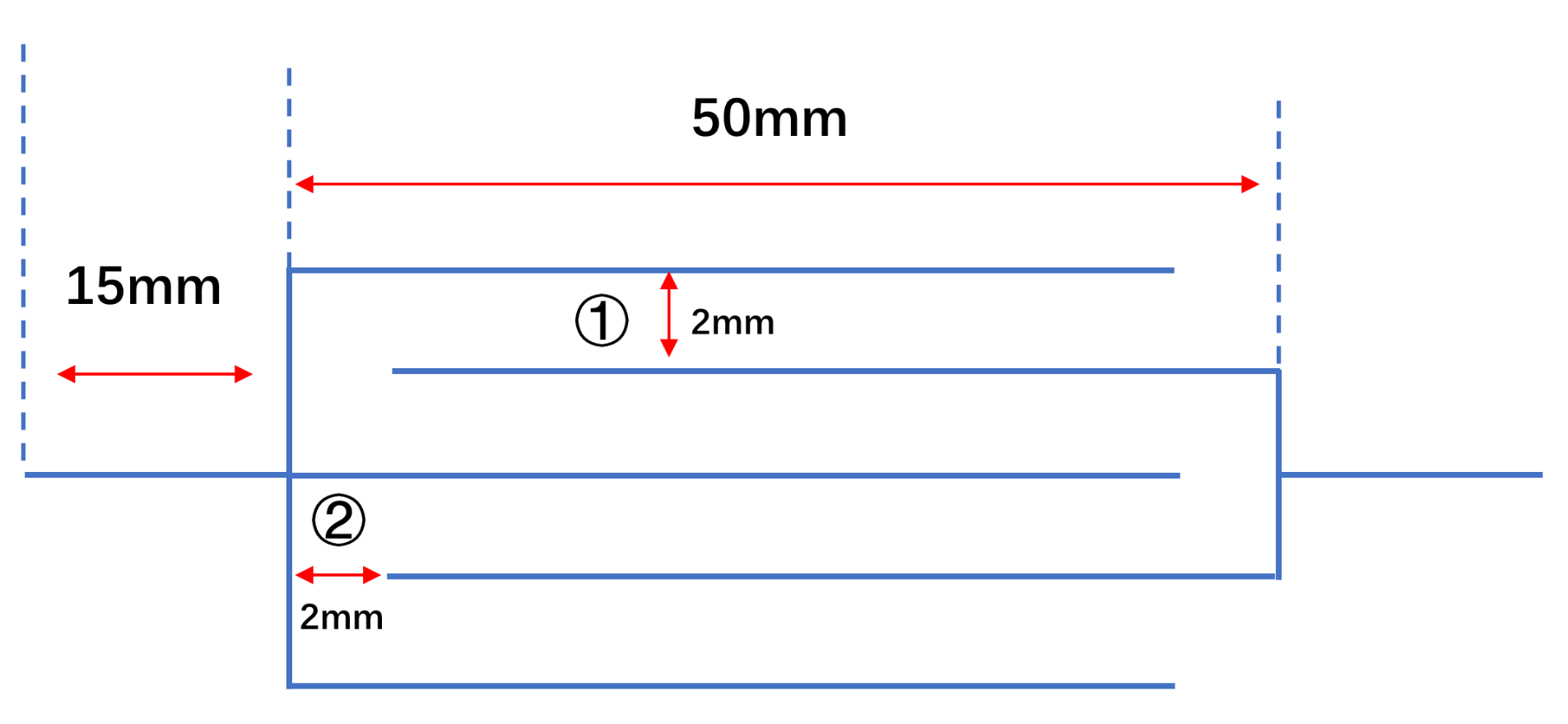


Figure S33. Circuit diagram of a Capacitive-based sensor (PCBS).


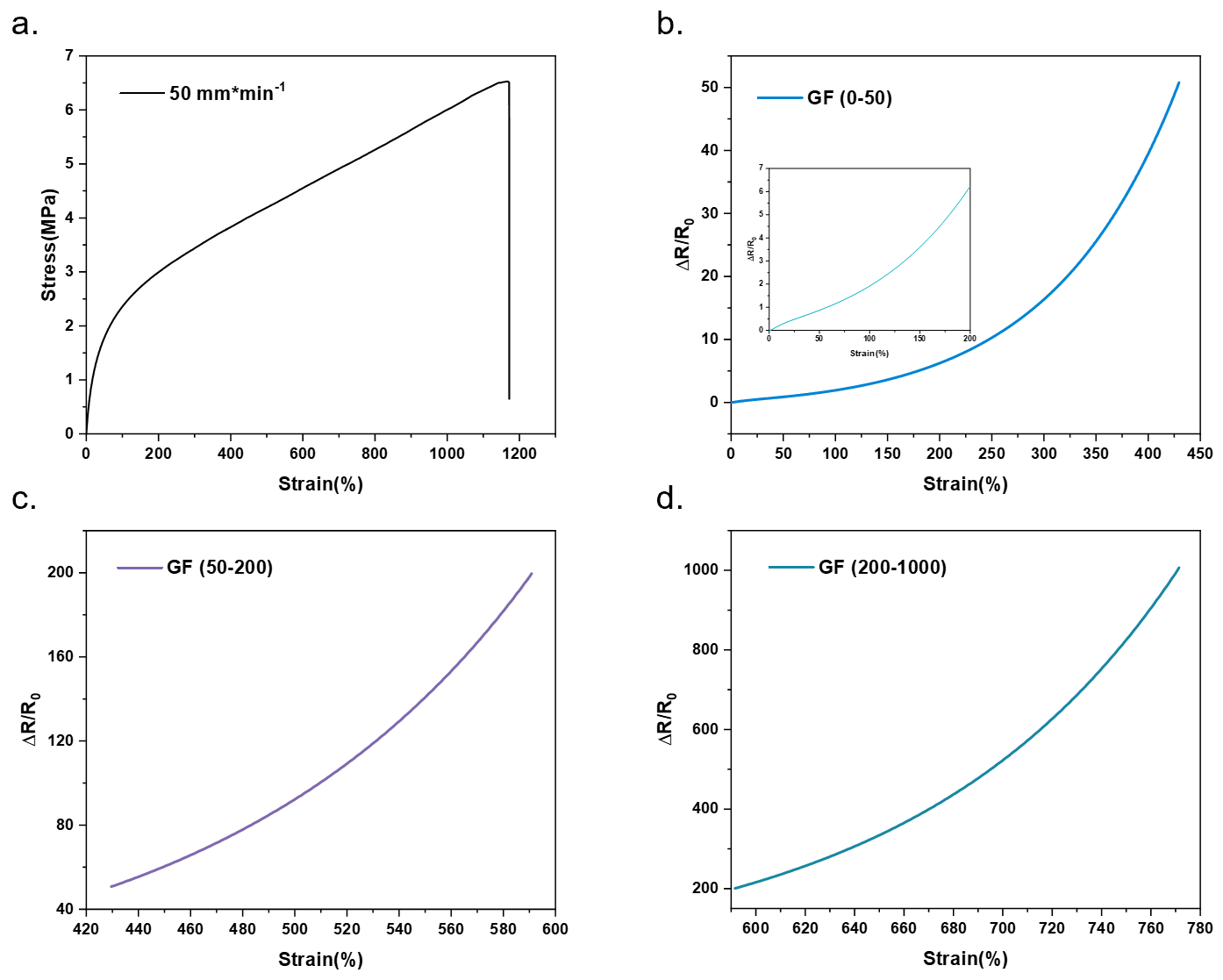


Figure S34. (a) Mechanical properties of SPPU-3_10%CB_. (b)-(d) Resistance change (ΔR/R_0_) of SPPU-3_10%CB_ during stretching process.


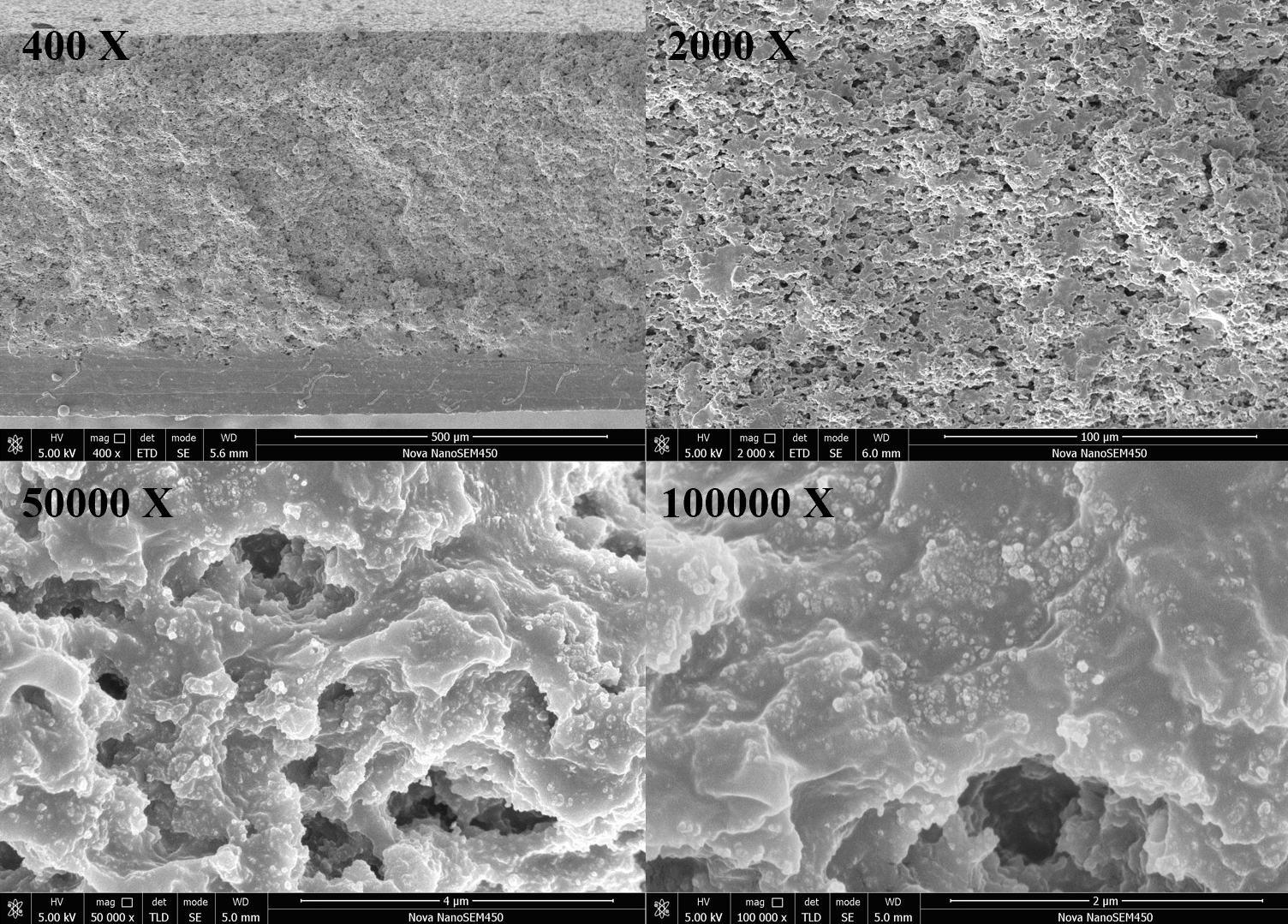


Figure S35. SEM image of SPPU-3_10%CB_.


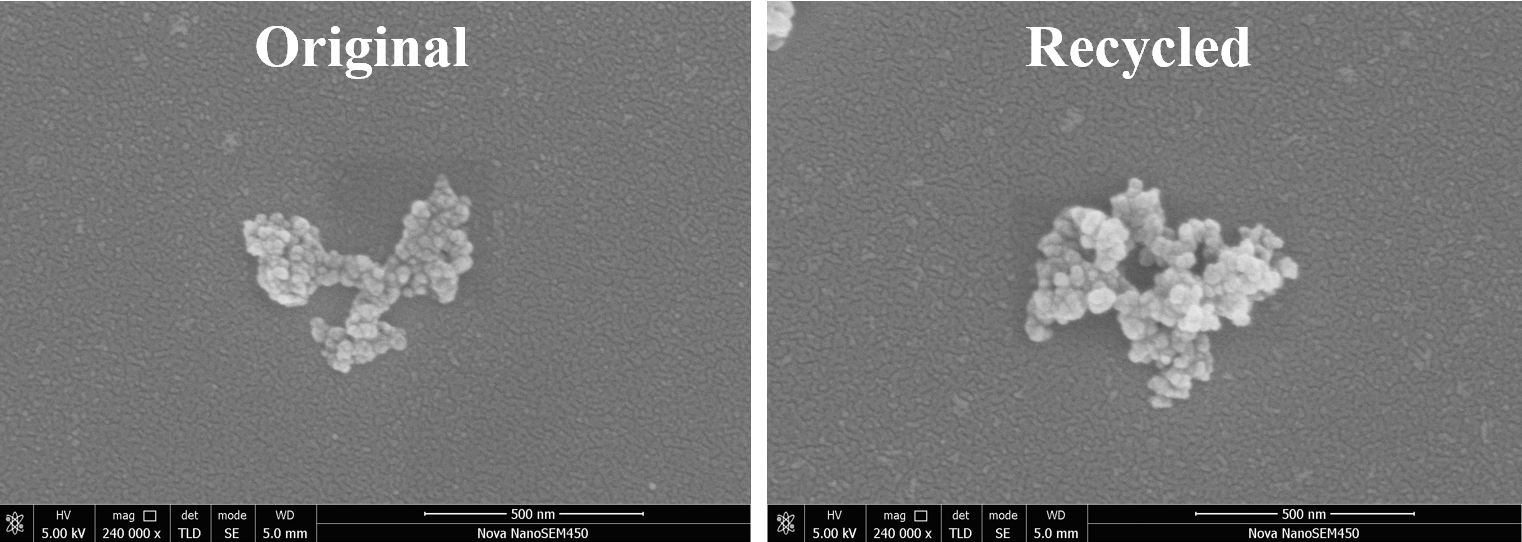


Figure S36. SEM images of the original and recovered carbon blacks.

We modified the filler from Ni powder to carbon black (CB), which was blended with an ethanol solution of SPPU-3 to obtain SPPU-3_10%CB_. Subsequently, a transient circuit was designed. Figure S34a present the mechanical properties curve of SPPU-3_10%CB_. From SEM images in Figure S35, it can be clearly seen that there are many holes in SPPU-3_10%CB_, resulting in obvious mechanical property degradation. Moreover, the high molecular weight of SPPU-3 causes poor stretching of molecular chain in ethanol, which in turn induces poor dispersion of carbon black in the ethanol solution of SPPU-3 and the aggregation phenomenon. The synergistic effect of the above two results in a significant decrease in the mechanical properties of SPPU-3_10%CB_. However, its mechanical properties are still favorable, possessing a high elongation at break (>1000%), which enables it to fulfill the requirements for the preparation of transient circuits. Figure S34 b-d show the point signal changes of SPPU-3_10%CB_ during stretching. Based on the alcohol-soluble property of SPPU-3, we designed a transient circuit. The circuit consists of a power supply, LEDs and SPPU-3_10%CB_, the bulb lights up when a pathway is formed, otherwise the bulb dies out. As shown in Video 1, the bulb lights up in the initial state, and when SPPU-3_10%CB_ is immersed in ethanol, the brightness of the bulb decreases significantly in about 8 s, and turns off completely about 15 s. This phenomenon indicates that the excellent alcohol solubility of SPPU-3_10%CB_ enables it to be promisingly used in transient recyclable circuits. Notably, high-speed centrifugation of dissolved SPPU-3 enabled the recovery of carbon black. As shown in Figure S36, analogous to SPPU-31/3Ni, it was initially confirmed by SEM that the morphology of carbon black before and after recovery remained consistent, indicating that our proposed method can be applied in a variety of systems.

**5. Videos**

**Video 1** The product application of transient electronic devices.

The process is that the bulb lights up in the initial state, but the brightness of the bulb decreases significantly in about 8 s when SPPU-310%CB is immersed in ethanol, finally turns off completely about 15 s.

**References**

[1] K. Zheng, F. Gu, H. Wei, L. Zhang, X. a. Chen, H. Jin, S. Pan, Y. Chen, S. Wang, *Small Methods* **2023**, *7* (4), <https://doi.org/10.1002/smtd.202201534>.

[2] Y. Su, J. Zhao, W. Zhan, H. Yuan, L. Wu, G. Sui, H. Zhang, *Chem. Eng. J.* **2022**, *435*, <https://doi.org/10.1016/j.cej.2022.135018>.

[3] J. Xiao, T. Zhu, H. Zhang, W. Xie, R. Dong, Y. Li, X. Wang, *Angew. Chem. Int. Edit.* **2024**, *n/a* (n/a), e202411270, <https://doi.org/https://doi.org/10.1002/anie.202411270>.

[4] R. Qin, M. Hu, X. Li, T. Liang, H. Tan, J. Liu, G. Shan, *Microsystems & Nanoengineering* **2021**, *7* (1), 100, <https://doi.org/10.1038/s41378-021-00327-1>.

[5] S. Liu, Y. Li, J. Wen, Z. Shen, Q. Meng, Q. Liu, F. Yang, S. Yu Zheng, J. Li, Z. Sun, G. Zhuang, J. Yang, *Adv. Funct. Mater.* **2024**, *34* (16), 2313397, <https://doi.org/https://doi.org/10.1002/adfm.202313397>.

[6] R. Ji, S. Yan, Z. Zhu, Y. Wang, D. He, K. Wang, D. Zhou, Q. Jia, X. Wang, B. Zhang, C. Shi, T. Xu, R. Wang, R. Wang, Y. Zhou, *Advanced Science* **2024**, *n/a* (n/a), 2401869, <https://doi.org/https://doi.org/10.1002/advs.202401869>.

[7] X. Huang, Z. Zheng, H. Wang, W. Xu, M. Wu, M. Wang, C. Chen, L. Wan, R. Du, T. Zhu, Z. Huang, X. Wang, X. Wang, Q. Zhang, X. Jia, *Adv. Funct. Mater.* **2024**, *34* (16), 2312149, <https://doi.org/https://doi.org/10.1002/adfm.202312149>.

[8] S. Chen, Y. Wang, L. Yang, F. Karouta, K. Sun, *Nano-Micro Letters* **2020**, *12* (1), 136, <https://doi.org/10.1007/s40820-020-00480-8>.

[9] Y. Guo, L. Yang, L. Zhang, S. Chen, L. Sun, S. Gu, Z. You, *Adv. Funct. Mater.* **2021**, *31* (50), 2106281, <https://doi.org/10.1002/adfm.202106281>.

[10] G. Yun, S.-Y. Tang, S. Sun, D. Yuan, Q. Zhao, L. Deng, S. Yan, H. Du, M. D. Dickey, W. Li, *Nat. Commun.* **2019**, *10*, 1300 <https://doi.org/10.1038/s41467-019-09325-4>.
